# Supplementary material for: Uncovering the reaction mechanism behind CoO as active phase for CO2 hydrogenation
Source: Nat Commun. 2022 Jan 14;13:324. doi: 10.1038/s41467-022-27981-x (PMC8760247; doi:10.1038/s41467-022-27981-x)
Supplement: Supplementary file 1 — Supplementary Information [file 41467_2022_27981_MOESM1_ESM.pdf]

Supplementary Information for

Uncovering the Reaction Mechanism behind CoO as Active Phase for CO_2 Hydrogenation

Iris C. ten Have,[1] Josepha J.G. Kromwijk,[1] Matteo Monai,[1] Davide Ferri,[2] Ellen B. Sterk,[1] Florian Meirer,[1,*] and Bert M. Weckhuysen[1,*]

[1]Inorganic Chemistry and Catalysis Group, Debye Institute for Nanomaterials Science, Utrecht University, Universiteitsweg 99, 3584 CG Utrecht, The Netherlands.

[2]Paul Scherrer Institute, Forschungsstrasse 111, 5232 Villigen, Switzerland.

*e-mail: f.meirer@uu.nl, b.m.weckhuysen@uu.nl

Table of contents

1. Supplementary Methods
- 1.1. Catalyst preparation……2
- 1.2. *Operando* Raman micro-spectroscopy……2
- 1.3. H_2-temperature programmed reduction……2
- 1.4. CO_2-temperature programmed reduction……3
- 1.5. Density functional theory calculations……3
- 1.6. *Operando* modulation excitation diffuse reflectance infrared Fourier transform spectroscopy……4
- 1.7. Data analysis……6
- 1.8. Kinetic parameters for Co-TiO_2-ox and Co/TiO_2-red……6
- 1.9. Thermodynamic calculations……6

2. Supplementary Discussion
- 2.1. Particle size analysis with X-ray diffraction……7
- 2.2. *Operando* Raman micro-spectroscopy……7
- 2.3. H_2-temperature programmed reduction……13
- 2.4. CO_2-temperature programmed reduction……14
- 2.5. Catalytic performance……15
- 2.6. Density functional theory calculations……16
- 2.7. Modulation excitation diffuse reflectance infrared Fourier transform spectroscopy……18
- 2.8. Co-feeding CO during CO_2 hydrogenation and comparison of catalytic performance……25
- 2.9. Kinetic parameters for Co-TiO_2-ox and Co/TiO_2-red……25
- 2.10. Thermodynamics……26
- 2.11. Long-term stability testing for Co/TiO_2……27

Supplementary References

1. Supplementary Methods

1.1. Catalyst preparation

The BET surface areas and pore volumes of the support materials were determined from N_2 adsorption-desorption isotherms measured at -196°C using a Micromeritics TriStar II PLUS instrument. Prior to the physisorption measurements, the samples were dried at 300°C overnight in N_2 flow. The resulting physical properties of the support materials and the exact amounts of chemicals used during the incipient wetness impregnation (IWI) preparation procedure can be found in **Supplementary Table 1**. All support oxides were purchased from companies (**Supplementary Table 1**), apart from CeO_2, which was synthesized in-house by homogeneous deposition precipitation (HDP) using urea as a precipitation agent. In 1.7 L of deionized water, 50 g cerium (III) nitrate hexahydrate (99.99% trace metals basis, Sigma-Aldrich) and 27 g of urea were dissolved. The mixture was added to a double-walled glass vessel and heated to 90°C for ~20 h while stirring at 600 rpm. The sample was washed three times with 500 mL deionized water, dried at 60°C, and then at 120°C. Calcination was performed at 500°C for 2 h (5°C min^{-1} ramp).

Supplementary Table 1. Support properties and amounts of precursor, support, and water used for IWI catalyst synthesis.

Support material	Supplier	Purity	BET surface area (m_2 g^{-1})	Pore volume (cm_3 g^{-1})	$Co(NO_3)_2{\cdot}6H_2O$ (g)	Support (g)	Water (mL)
SiO_2 (Davisil grade 643)	Sigma-Aldrich	≥99%	300	1.15	0.981	1.812	2.0
γ-Al_2O_3	CRI Catalyst Company	≥99%	262	1.03	0.949	1.818	2.0
TiO_2 (P25)	CRI Catalyst Company	≥99%	52.7	0.31	0.991	1.815	0.6
CeO_2	n.a.	n.a.	88.5	0.06	0.949	1.792	0.3

1.2. *Operando* Raman micro-spectroscopy

The *operando* Raman micro-spectroscopy experiments were carried out with a Horiba Xplora Raman microscope equipped with a 532 nm laser (output of 0.53 MW), a 1200 nm grating and a 50x objective. The range was set to 100-2800 cm^{-1}, the acquisition time was 10 s and 5 accumulations were performed. The catalyst sample was placed in a THMS600 cell from Linkam Scientific Instruments. The Linkam cell was connected to a Bronkhorst mass flow controller (MFC) for gas input. The output of the cell was connected to an OmniStar mass spectrometer from Pfeiffer Vacuum to analyze the gaseous products. In a typical experiment about 10-15 mg of catalyst sample was placed in the Linkam cell. The temperature was ramped to 250°C (suffix: -ox) or 450°C (suffix: -red) with 10°C min^{-1} under a gas mixture of Ar:H_2 40:20 mL min^{-1} and held there for 1 h. Then, the cell was either held at or cooled down to 250°C and the gas flow was switched to Ar:H_2:CO_2 in a ratio of 40:10:2.5 mL min^{-1}. The temperature was held on this for one hour. During this hour, the gaseous products were continuously analyzed by the mass spectrometer. Raman spectra were collected before and during the reaction.

1.3. H_2-temperature programmed reduction (TPR)

H_2-TPR measurements were performed using a Micromeritics AutoChem II 2920. Samples were placed on quartz wool into a U-tube quartz reactor. The gas mixture consisted of 5% H_2 in Ar with a total gas flow of 40 ml min^{-1}. H_2-TPR was carried out by heating with 5°C min^{-1} up to 1000°C for all cobalt-based catalysts and held for

30 min at this temperature. A constant initial sample weight of 0.05 g was used and H_2 consumption was continuously monitored by a thermal conductivity detector. To assess reducibility, a centroid was calculated for each data set. This was done by calculating the integral (MATLAB) of the H_2-TPR data for each sample and then taking ½ of that.

1.4. CO_2-temperature programmed desorption (TPD)

CO_2 Temperature Programmed Desorption (TPD) measurements were carried out on a Micrometrics ASAP2920 instrument equipped with a thermal conductivity detector. In a typical experiment, 100 mg of sample (SiO_2, Al_2O_3, TiO_2, or CeO_2; see **Supplementary Table 1** for details) was loaded in a quartz tube and dried *in situ* by ramping with 5°C min^{-1} to 300°C in a He flow and remained at that temperature for 60 min. Subsequently, the sample was cooled down to 60°C; at this temperature pulses of 10% CO_2 in He of 25 cm^3/min were applied. After saturation with CO_2, the sample was outgassed for 30 min at 60°C to ensure removal of physisorbed CO_2. Finally, the sample was heated to 700°C with a ramp of 5°C min^{-1} to measure CO_2 desorption. To assess support basicity, the integral of the CO_2-TPD data was calculated for each data set and divided by the BET surface area of the respective sample.

1.5. Density functional theory (DFT) calculations

Quantum-chemical calculations in this work were performed using a planewave density function theory (DFT) approach with the projector-augmented wave (PAW) method[1,2] in conjunction with a Perdew-Becke-Ernzerhof (PBE) exchange-correlation functional[3] as implemented in Vienna Ab-initio Simulation Package (VASP)[4,5]. The kinetic energy cutoff for the plane wave basis set was 400 eV. For all calculations spin polarization was considered explicitly. A conventional fcc-Co and fcc-CoO unit cell was used to build the surface terminations. Herein, the bulk lattice constant of Co and CoO in their face-centered cubic crystal (fcc) structure was optimized yielding a theoretical optimum of 3.34 Å and 4.265 Å respectively. These values correspond well to the documented bulk lattice constant of 3.42 Å for Co and 4.260 Å for CoO[6,7].

The Co(111) , Co(110) and CoO(100) surfaces were modeled using a (3x3) surface, with 6, 4 and 6 metal layers, respectively. A Monck-horst-Pack mesh of k-points of (5x5x1) for Co(110) and (3x3x1) for Co(111) and CoO(100) were used[8]. A vacuum layer of 15 Å perpendicular to the surface was employed to avoid the spurious interaction of neighboring supercells. To avoid the build-up of a large dipole moment between neighboring supercells, the adsorbates were placed on both sides of the surface slabs retaining a point of inversion. All atoms were allowed to relax. Partial occupancies were determined using a first-order Methfessel-Paxton scheme with a smearing width of 0.2 eV and 0.03 eV for the Co and CoO slabs, respectively[9]. Electronic convergence was set to 10^{-5} eV, and geometries were converged to 10^{-4} eV using a conjugant gradient algorithm for the Co systems and a quasi-Newton algorithm for CoO system. For the gas-phase calculation of CO_2, the molecule was placed in a 10x10x10 Å unit cell. Gaussian smearing with a width of $2x10^{-5}$ eV was used for electron smearing and only the gamma-point was used to sample the Brillouin zone.

The adsorption energy, E_{ads}, is defined as follows:

$$E_{\text{ads}} = E_{\text{slab+adsorbate}} - E_{\text{slab}} - E_{\text{adsorbate}}$$

where $E_{\text{slab+adsorbate}}$ represents the total energy of the optimized adsorbate on the surface, E_{slab} is the energy of the nickel slab and $E_{\text{adsorbate}}$ is the energy of the adsorbate in the gas phase. The total density of states was calculated in the energy range of -30 eV to 15 eV over 4500 grid points. Then, the partial charge density corresponding to the energy interval of each molecular orbital was calculated. The contour plots of the electron

density from these intervals were plotted on a cutting plane running parallel through CO_2 adsorbed on the top of the slab.

1.6. *Operando* modulation excitation (ME) diffuse reflectance infrared Fourier transform spectroscopy (DRIFTS)

A picture of the *operando* modulated excitation (ME) diffuse reflectance infrared Fourier transform spectroscopy (DRIFTS) setup and a schematic drawing of the cell used during the experiments can be found in **Supplementary Figure 1.** The cell was designed to represent a plug flow reactor and has a reduced dead volume, which allows for fast exchange of the gas atmosphere and ensures suitability for transient experiments[10]. The gas flows were controlled with mass flow controllers (MFCs), which were calibrated in advance. During the modulation experiments, the gas inlet was controlled with fast switching valves (solenoid valves) that were operated using an automated script. The experiments were carried out as graphically represented in in **Supplementary Figure 2** by admitting to the catalyst sample equally long (60 s) pulses of CO_2/H_2 and H_2 (modulation period, H_2/CO_2=3) at the selected temperature 250°C while recording 120 spectra (1 spectrum/s) at 80 kHz scanner velocity and at 4 cm^{-1} resolution. One experiment consisted of 10 modulation periods. Prior to the modulation experiments, the sample (typically 35-40 mg with a grain size of 250-63 μm) was heated to 250°C at 10°C min^{-1} in H_2/N_2 (H_2/N_2=1; total flow rate 40 mL min^{-1}) to create the CoO oxidation state. Then, at 250°C 10 modulation periods were performed by alternating flows of CO_2:H_2 (H_2/CO_2 =3; 6 mL min^{-1} H_2, 2 mL min^{-1} CO_2, 12 mL min^{-1} N_2 total flows) and H_2 (6 mL min^{-1} H_2 and 12 mL min^{-1} N_2 total flows). During each period of 120 s, 120 spectra (1000-4000 cm^{-1} with 4 cm^{-1} spectral resolution) were recorded. The 10 modulation periods of 120 s each resulted in a 20 min experiment. After the modulation experiment, the sample was heated to 450°C at 10°C min^{-1} in H_2/N_2 (H_2/N_2=1; total flow rate 40 mL min^{-1}) and was held there for 1 h to reduce the CoO nanoparticles to metallic Co. Then, the sample was cooled to 250°C at 10°C min^{-1} and the modulation experiment described above was repeated. During the experiments, the gaseous products were analyzed using an Omnistar quadrupole mass spectrometer from Pfeiffer Vacuum using the following m/z values: 2 (H_2), 4 (helium), 14 (methane), 15 (methane), 18 (water), 28 (CO or ethylene), 32 (methanol), 40 (argon), 44 (CO_2), 26 (acetylene), 29 (C_{2+} hydrocarbons), 43 (C_{2+} hydrocarbons), 45 and 46 (ethanol).

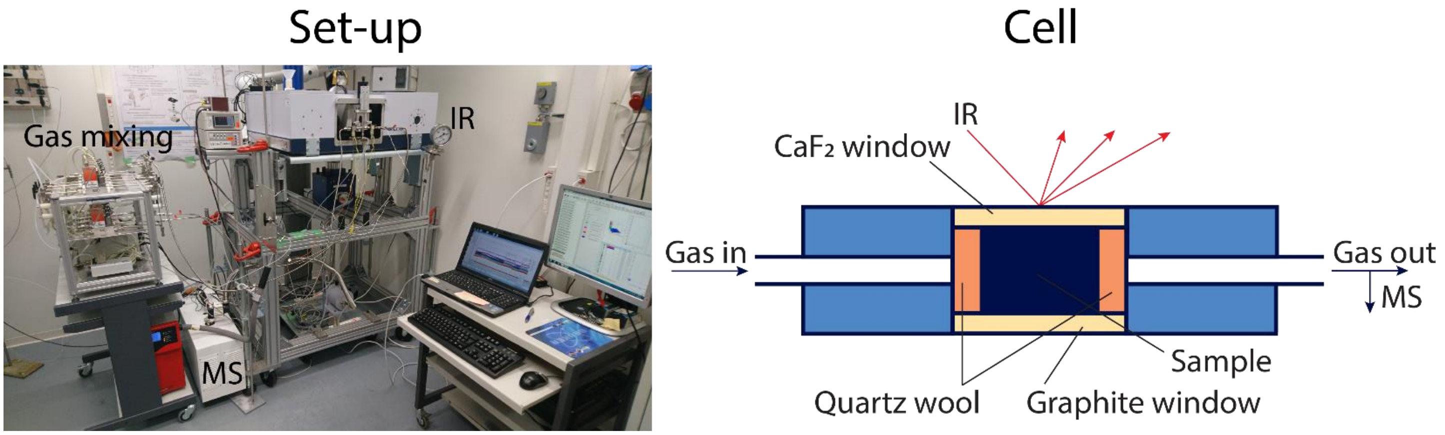

Supplementary Figure 1. Picture of the ME DRIFTS setup (left), showing the gas mixing panel, the mass spectrometer (MS) for product analysis, and the infrared (IR) spectrometer equipped for diffuse reflectance infrared Fourier transform spectroscopy (DRIFTS). On the right a schematic drawing of the cell used in this study. More details can be found in reference [10].

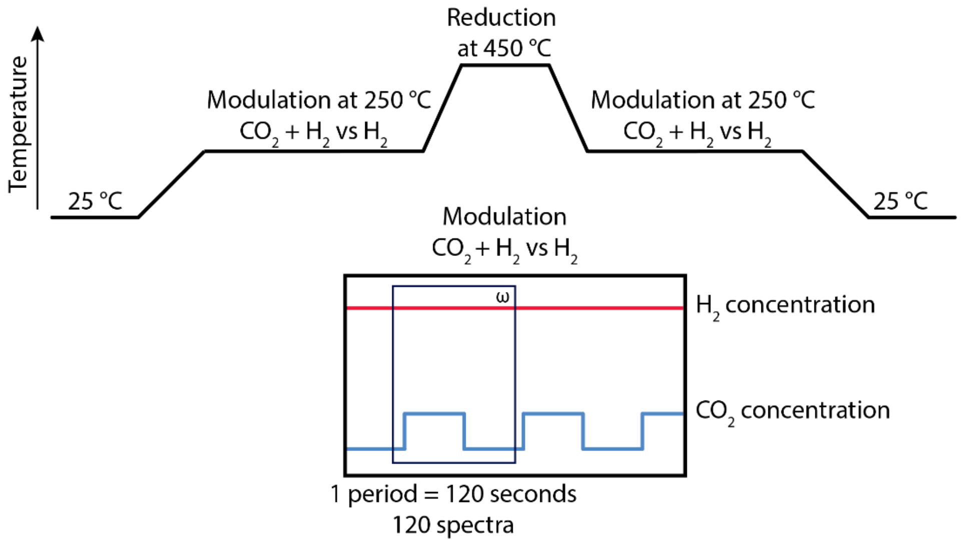

Supplementary Figure 2. Graphical representation of the ME DRIFTS experimental procedure.

The working principle of modulated excitation spectroscopy is represented in **Supplementary Figure 3**. This procedure is similar to a lock-in amplifier, where a weak signal is filtered out of a noisy background[11]. Phase sensitive detection (PSD, also termed demodulation) of the time-resolved data, *i.e.* the translation from the time domain to the phase domain, yields the data in **Supplementary Figure 10**.

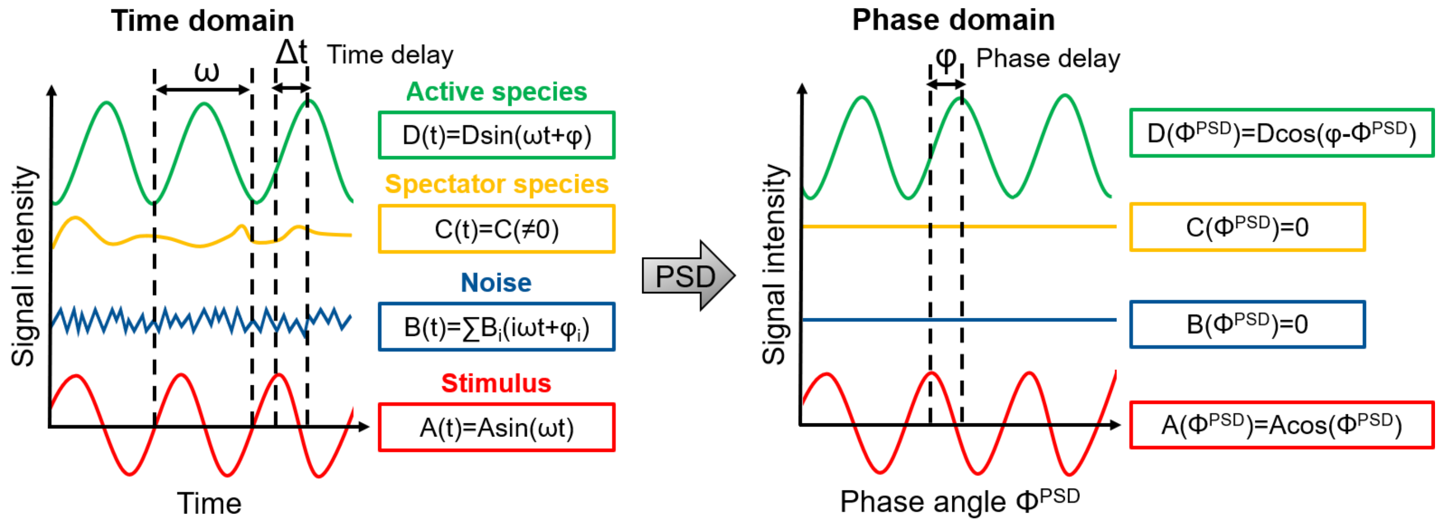

Supplementary Figure 3. Schematic overview of the principle of phase-sensitive detection (PSD). $A(t)$ is the external stimulus, $B(t)$ is noise, $C(t)$ is the response of a spectator species, and $D(t)$ is the response of an active species. The external stimulus is a sinusoidal or square-wave function with frequency ω. Once the full set of time-resolved data (time domain) is measured along a given number of modulation periods, each spectrum is averaged along the modulation periods to obtain a set of average time-resolved spectra (spectrum i is the average of spectrum i from each modulation period)[12]. Demodulation by PSD transforms the set of averaged time-resolved data into a set of phase-resolved data (phase domain). In this set of data, the active species appear with a phase delay ϕ instead of appearing with a time delay Δt. The phase delay contains additional kinetic information about the system that is often hidden in the kinetic information delivered by the time-resolved data (for example because of strong band overlap). The phase-resolved amplitude spectra in **Figure 2b** in the

main text were obtained by taking the absolute maximum at every single wavenumber in the phase-resolved spectra. The result is that only positive signals are displayed for the sake of clarity in the discussion.

1.7. Data analysis

The spectra were analyzed using LabSpec6 (Raman), MATLAB (ME DRIFTS), and Origin 9.

1.8. Kinetic parameters for Co-TiO_2-ox and Co/TiO_2-red

The kinetic parameters reaction order (*n*, *m*) and apparent activation energy (E_a) were determined by varying the reactant (CO_2, H_2) concentration and the temperature, respectively. For these experiments the high-pressure catalytic testing setup, as described in the Methods section of the main text, was used. The catalyst was either treated at 250°C in H_2 for 1 h to obtain the CoO phase (Co/TiO_2-ox) or at 450°C in H_2 for 1 h to obtain the metallic Co phase (Co/TiO_2-red). Then, at *T*=250°C and *P*=20 bar, the H_2/CO_2 ratio was varied at 2, 3, and 4 while the H_2 concentration was kept constant and the H_2/CO_2 ratio was varied at 2 and 3 while the CO_2 concentration was kept constant. This was repeated three times in order to obtain standard deviations. The measured intrinsic reaction rates were used to calculate the reaction order (*n*) in CO_2 and (*m*) in H_2 according to the rate law (**Supplementary Equation** (**1**))[13], where *k* is the rate constant, *n* the reaction order in CO_2, and *m* the reaction order in H_2. Besides, the temperature was varied between 200 and 280°C at *P*=20 bar and the measured intrinsic rates were used to calculate the apparent activation energy (E_a) according to the Arrhenius equation (**Supplementary Equation** (**2**))[13], where *k* is the rate constant, *A* is the pre-exponential factor, *R* the universal gas constant, and *T* the absolute temperature in Kelvin.

$$\mathrm{rate} = k[\mathrm{CO_2}]^n[\mathrm{H_2}]^m \quad (\mathbf{1})$$

$$k = A\mathrm{e}^{-E_\mathrm{a}/RT} \quad (\mathbf{2})$$

1.9. Thermodynamic calculations

Thermodynamic calculations were performed using the software HSC 9.6.1, in the Gem equilibrium composition module, based on Gibbs free energy minimization[14].

2. Supplementary Discussion

2.1. Particle size analysis with X-ray diffraction (XRD)

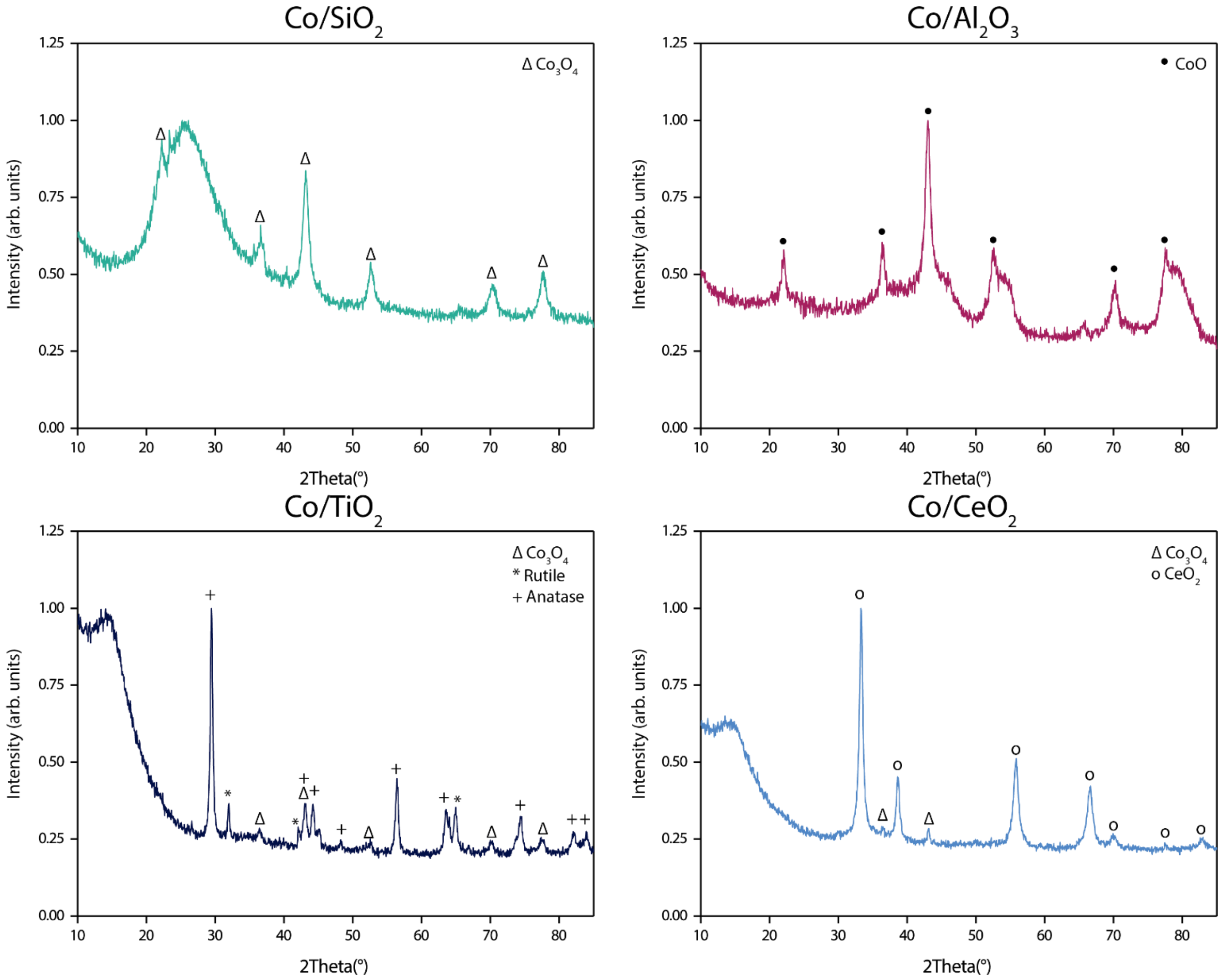

Supplementary Figure 4. X-ray diffractograms of the calcined (250°C; N_2) cobalt catalysts as prepared by incipient wetness impregnation (IWI).

Supplementary Table 2. Average crystallite size of cobalt particles on various supported cobalt catalysts. The diffraction peak at 43° was used to determine the crystallite size using the Scherrer equation in the Bruker EVA software.

Catalyst	Cobalt phase	Average crystallite size (nm)
Co/SiO_2	Co_3O_4	12.5
Co/Al_2O_3	Co_3O_4	13.2
Co/TiO_2	Co_3O_4	14.6
Co/CeO_2	Co_3O_4	30.7

2.2. *Operando* Raman micro-spectroscopy

Operando Raman micro-spectroscopy was used to study the oxidation state of cobalt (see **Supplementary Figure 5**). Co_3O_4 has a peak at 690 cm^{-1}, whereas a CoO peak is observed at 675 cm^{-1}, and the vibrations of metallic cobalt are not observable, as they are Raman-inactive[15]. The catalysts treated at 250°C in H_2 (suffix: -ox) contained primarily CoO at 250°C during the CO_2 hydrogenation reaction (**Supplementary Figure 6**), as

indicated by the peak positions around 675 cm^{-1}. The catalysts treated at 450°C in H_2 (suffix: -red) contained metallic cobalt during CO_2 hydrogenation at 250°C, as indicated by the absence of a peak 675-690 cm^{-1}.

For the reducible supports, TiO_2 and CeO_2, (partial) reduction of the support was observed in the Raman spectra. For example, CeO_2 displayed a Ce^{4+}-O-Ce^{4+} wagging vibration at 458 cm^{-1} prior to the reaction. The CeO_2 lattice expands slightly upon reduction, as Ce^{3+} is larger than Ce^{4+}, causing the wagging vibrational frequency to shift down[16]. We observed indeed a shift from 458 cm^{-1} in the fresh Co/CeO_2 catalyst to *e.g.* 452 cm^{-1} during CO_2 hydrogenation at 250°C for Co/CeO_2-ox. A similar effect was observed for TiO_2, as *e.g.* the peak at 519 cm^{-1} (E_g)[17] prior to reaction shifted to 513 cm^{-1} during CO_2 hydrogenation at 250°C for Co/TiO_2-ox.

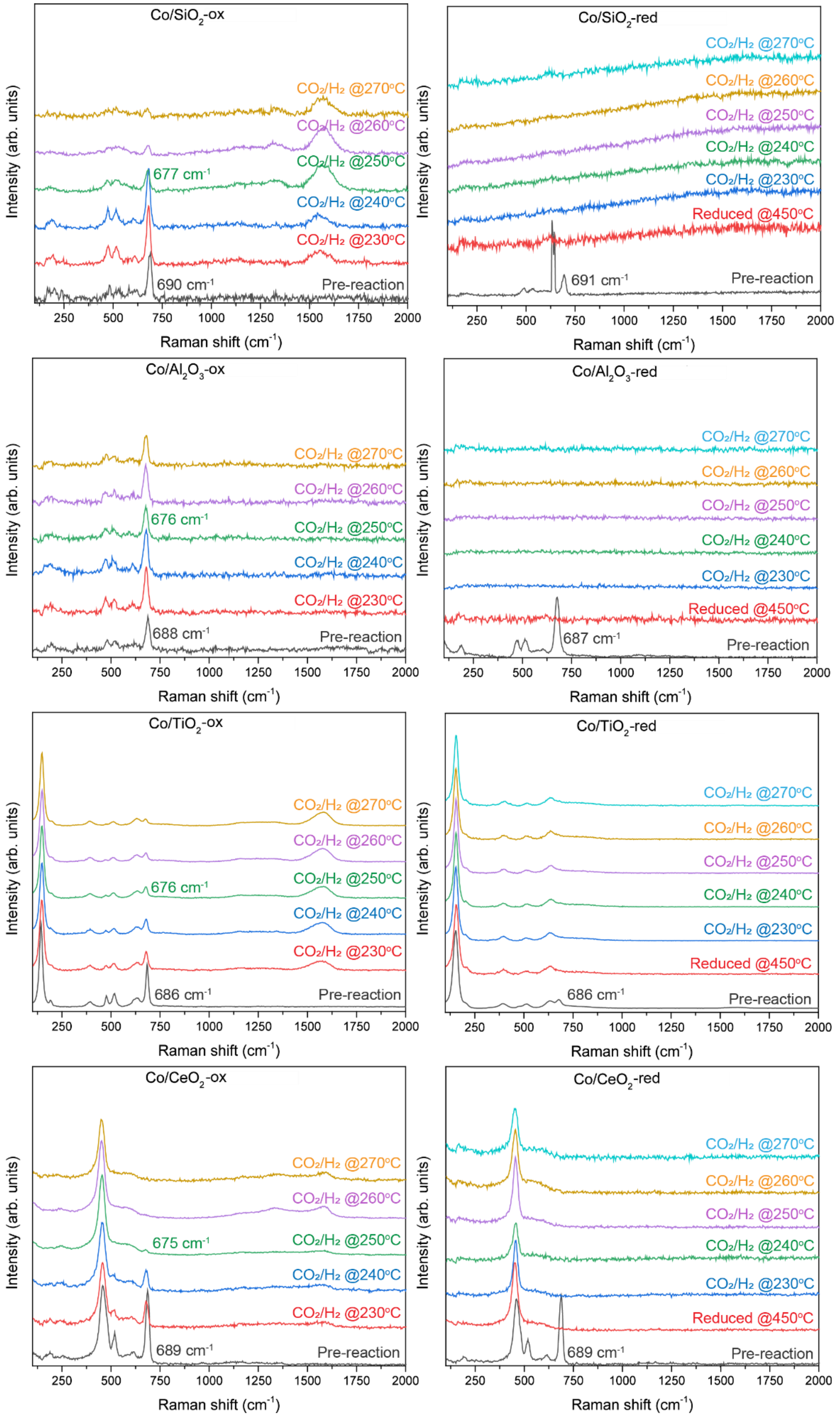

Supplementary Figure 5. *Operando* Raman spectra during CO_2 hydrogenation (H_2/CO_2=3) over the cobalt catalysts used in CoO (suffix: -ox, left) and metallic Co (suffix: -red, right) state. The reaction was carried out at ambient pressure and at temperatures between 230-270°C.

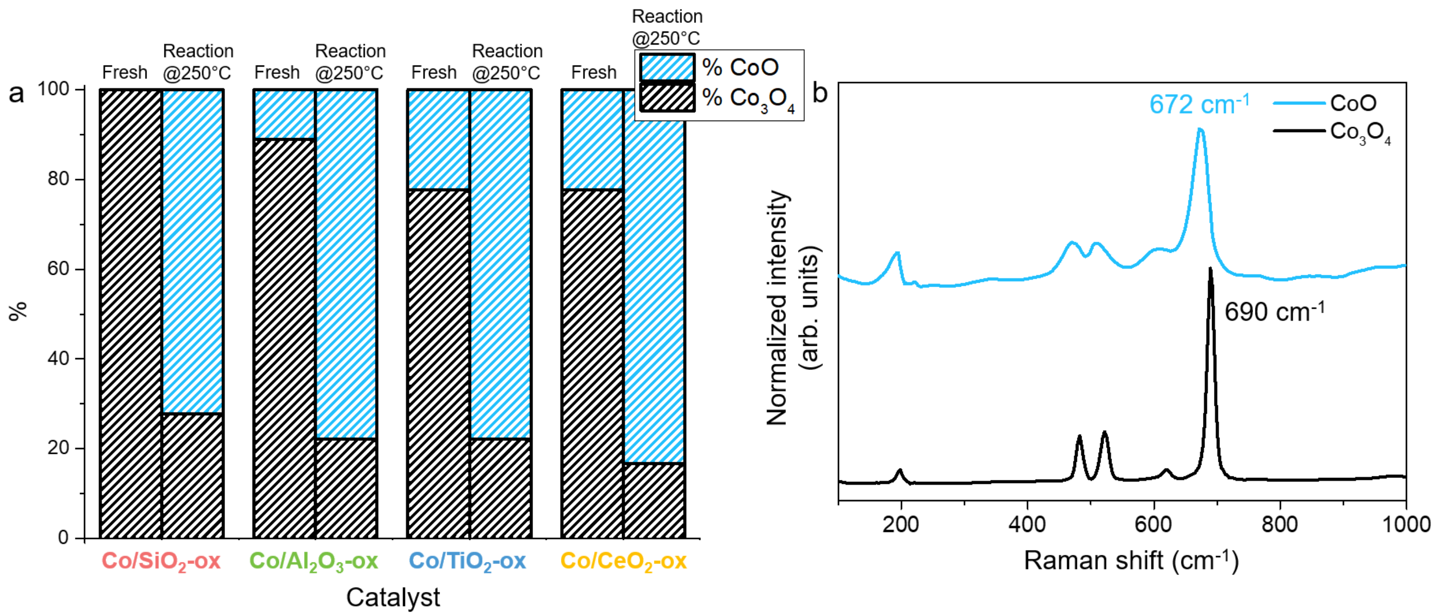

Supplementary Figure 6. a Percentages of CoO and Co_3O_4 present in the fresh cobalt-based catalysts and the catalysts pre-treated in Ar/H_2=4 at 250°C (suffix: -ox) under CO_2 hydrogenation reaction conditions (T=250°C, P=1 bar, H_2/CO_2=3) derived from peak positions in the *operando* Raman spectra. **b** Raman reference spectra of CoO (Sigma-Aldrich, 99.99% trace metal basis) and Co_3O_4 (Sigma-Aldrich, ≥99.99% trace metal basis). N.B. metallic Co is Raman inactive.

Supplementary Table 3. Raman peak maxima and full width at half maximum (FWHM) of cobalt-based catalysts before and during the CO_2 hydrogenation reaction at 250°C. Reference compounds Co_3O_4 and CoO are also included in the table and were used to calculate the percentages of Co_3O_4 and CoO in the catalysts.

Catalyst	Oxidation state	Peak maximum fresh (cm^{-1})	% Co_3O_4 fresh	% CoO fresh	FWHM fresh (cm^{-1})	Peak maximum reaction @250°C (cm^{-1})	% Co_3O_4 reaction @250°C	% CoO reaction @250°C	FWHM reaction @250°C (cm^{-1})
Co/SiO_2	CoO	690	100	0	24.6	677	27.8	72.2	27.7
Co/SiO_2	Metallic Co	690	100	0	24.9	n.a.	0	0	n.a.
Co/Al_2O_3	CoO	688	88.9	11.1	25.9	676	22.2	77.8	32.6
Co/Al_2O_3	Metallic Co	687	83.3	16.7	30.5	n.a.	0	0	n.a.
Co/TiO_2	CoO	686	77.8	22.2	16.4	676	22.2	77.8	28.0
Co/TiO_2	Metallic Co	686	77.8	22.2	18.0	n.a.	0	0	n.a.
Co/CeO_2	CoO	686	77.8	22.2	22.6	675	16.7	83.3	29.3
Co/CeO_2	Metallic Co	688	88.9	11.1	21.6	n.a.	0	0	n.a.
Co_3O_4	n.a.	690	100	0	13.8	n.a.	n.a.	n.a.	n.a.
CoO	n.a.	672	0	100	33.6	n.a.	n.a.	n.a.	n.a.

Besides, for the CoO catalysts (left side in **Supplementary Figure 5**) carbon deposition was observed during the CO_2 hydrogenation reaction, as indicated by the appearance of the D and G bands in the Raman spectra, typically found between 1200 and 1600 cm^{-1}[18]. The formation of carbonaceous materials on the catalyst surface, could lead to catalyst deactivation[19,20]. From the mass spectra recorded during the *operando* Raman experiments (**Supplementary Figure 7**), the CO_2 signal (m/z=44, orange line) indeed seemed lower for the CoO catalysts

compared to the metallic Co catalysts. Carbon deposition on the CoO catalysts could thus be (one of) the reason(s) for their lower activity. Though, for all catalysts the CO_2 signal decreased with temperature, indicating that more CO_2 was converted at higher temperatures. Besides, methane (m/z=16, magenta line) and H_2O (m/z=18, purple line) were followed during the *operando* Raman experiments. Their increase indicates increasing activity. The hydroxymethyl fragment (m/z=31, green line) was followed as a measure for oxygenated products. However, the hydroxymethyl fragment remained constant at 0 during all experiments and no prove was found for the formation of oxygenates. Small amounts of ethane or CO were indicated by the variation of the m/z=28 fragment (blue line in **Supplementary Figure 7**).

Activation energies (E_a) were calculated *via* Arrhenius plots from the CO_2 conversion[21] as measured with mass spectrometry at different temperatures during the *operando* Raman experiments (**Supplementary Figure 8**). For the CoO catalysts, the activation energy decreased with increasing support reducibility in the order of Co/SiO_2>Co/Al2O_3>Co/TiO_2>Co/CeO_2. For the metallic Co catalysts, the activation energies were typically lower compared to their CoO counterpart, except from Co/CeO_2, where Co/CeO_2-red had a higher activation energy than Co/CeO_2-ox.

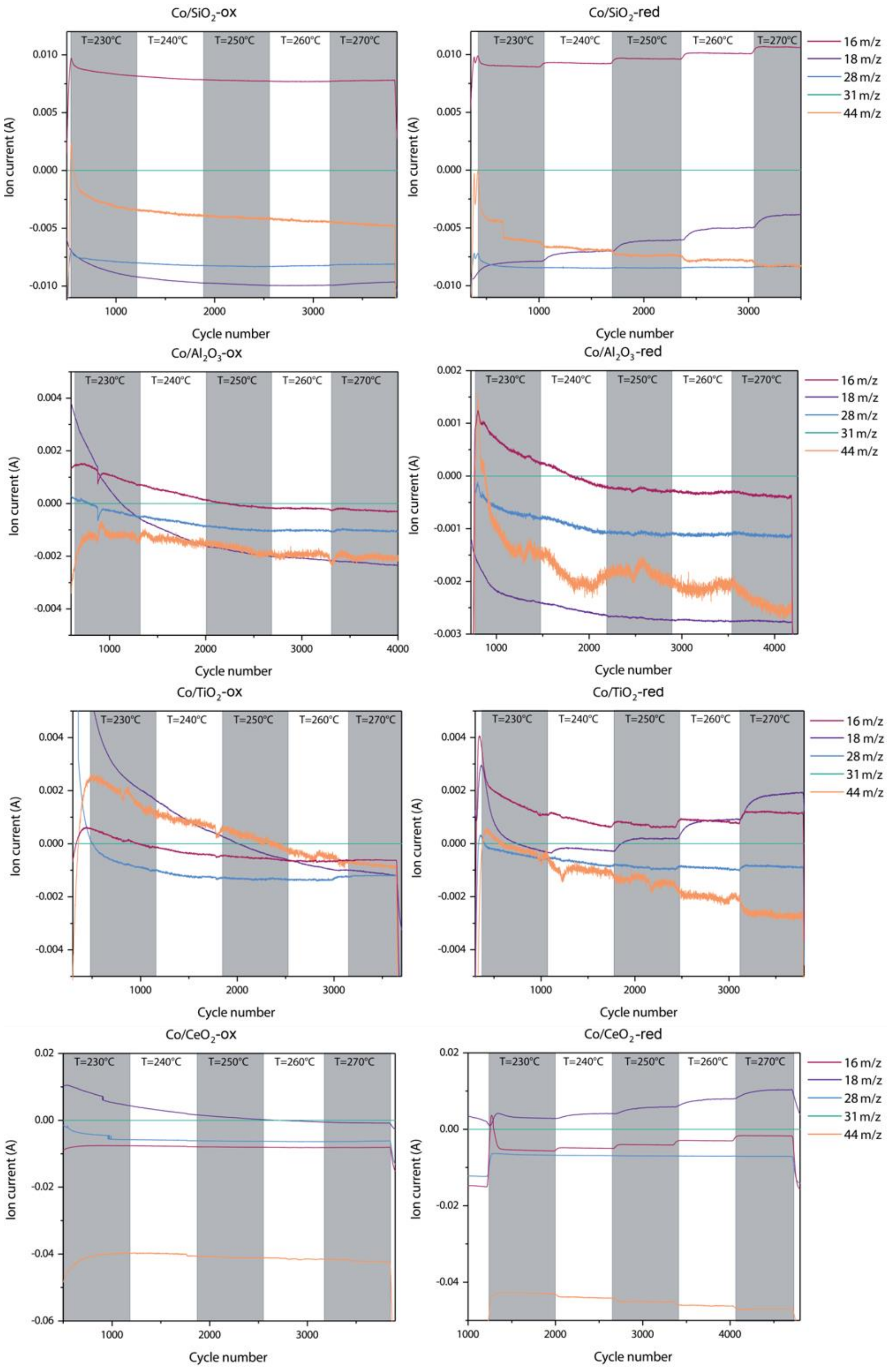

Supplementary Figure 7. Mass spectrometry results recorded during the *operando* Raman experiments in **Supplementary Figure 5**. The CO_2 hydrogenation reaction (H_2/CO_2=3) was carried out at ambient pressure and at temperatures between 230-270°C. The fragments plotted are methane (m/z=16), water (m/z=18), CO or ethylene (m/z=28), hydroxymethyl (m/z=31), and CO_2 (m/z=44). Suffix -ox denotes the CoO catalysts, pre-treated in Ar/H_2=4 at 250°C, and suffix -red denotes the metallic Co catalysts, pre-treated in Ar/H_2=4 at 450°C (suffix: -ox).

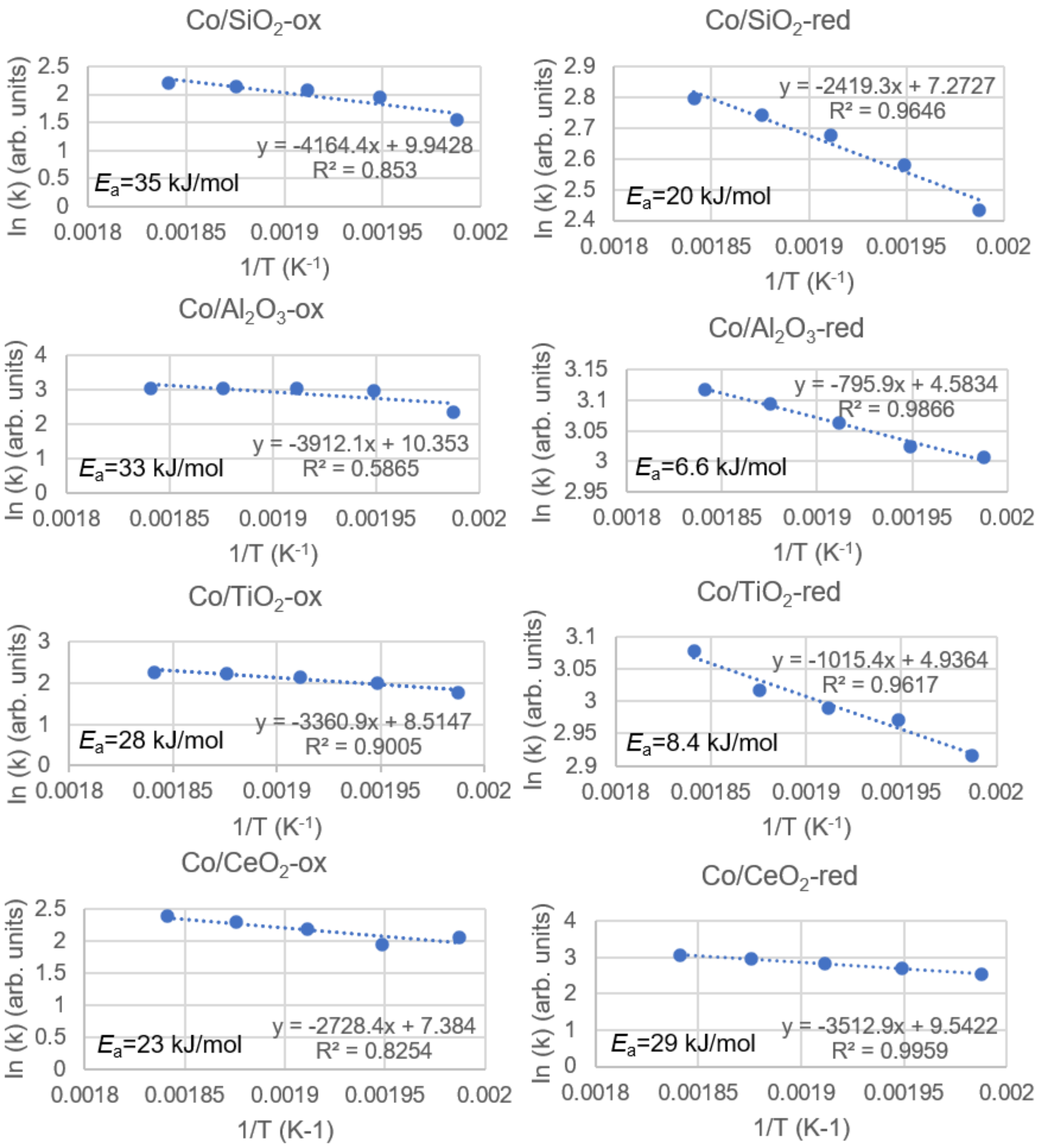

Supplementary Figure 8. Arrhenius plots and activation energies (E_a) for the CoO (left, suffix: -ox) and metallic Co (right, suffix: -red) catalysts. The values of k were calculated from the CO_2 conversion as measured with mass spectrometry during *operando* Raman experiments (**Supplementary Figures 5** and **7**).

2.3. H_2-temperature programmed reduction

The H_2-TPR profiles for Co/SiO_2, Co/Al_2O_3, Co/TiO_2, and Co/CeO_2 are displayed in **Supplementary Figure 10**. The first peak is typically assigned to the reduction of Co_3O_4 to CoO (@250-290°C), while the second peak is ascribed to the reduction of CoO to metallic Co (@300-420°C)[22]. For Co/Al_2O_3, two other peaks were clearly visible at 673 and 958°C, which have been ascribed to cobalt aluminates species. Similarly, for Co/SiO_2, an additional peak was observer around at 635°C, which could indicate the reduction of cobalt silicates[22,23].

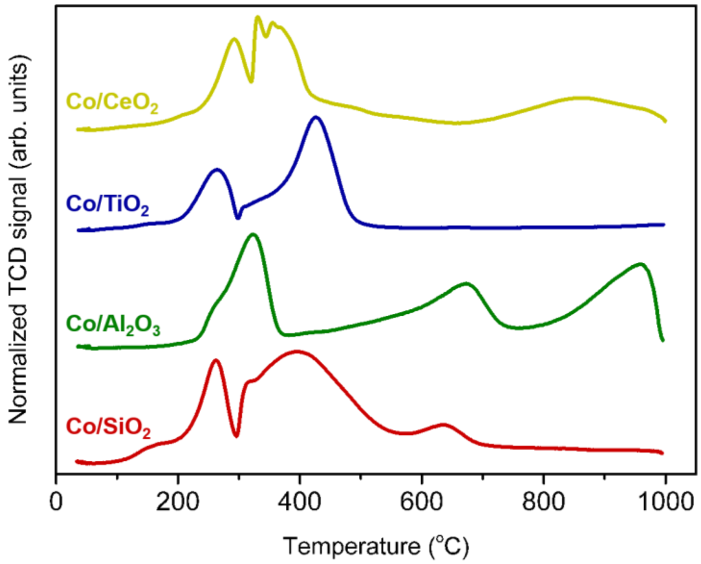

Supplementary Figure 9. H_2-TPR profiles of the cobalt-based catalyst under study recorded with a thermal conductivity detector (TCD).

2.4. CO_2-temperature programmed desorption

The CO_2-TPD profiles for the SiO_2, Al_2O_3, TiO_2, and CeO_2 support materials are displayed in **Supplementary Figure 10**. Different types of basic sites were identified: weak (<150°C), medium (150-350°C), and strong (>350°C)[24]. The TPD peaks generally shift to higher temperature and increase in intensity when the basic sites become stronger and increase in quantity[25]. Both the non-reducible supports SiO_2 and Al_2O_3 were dominated by strong basic sites[26], with their main desorption peaks at 430°C and 393°C, respectively. The reducible supports displayed different basic sites. For TiO_2, two peaks were observed: one at 199°C, which was assigned to bidentate carbonate decomposition from medium strength basic sites, and a smaller peak at 390°C, which was attributed to monodentate carbonate decomposition from strong basic sites. For CeO_2, we observed a peak at 135°C, ascribed to bidentate carbonate decomposition from weak-medium strength basic sites, and another peak at 397°C, interpreted as monodentate carbonate decomposition from strong basic sites[24]. The total amount of desorbed CO_2 was estimated from the integral of the CO_2-TPD peak area. The CO_2 surface density quantification was used as a measure for the basicity and increases from SiO_2<Al_2O_3<CeO_2<TiO_2 (**Supplementary Table 4**), which is conform with earlier reports in the literature[27,28]. Though, some studies report that CeO_2 was more basic compared to TiO_2[29,30], it has also been reported that the basicity decreased with increasing calcination temperature of the metal oxide[31] and is consequently a more complex cohesion than just the chemical element.

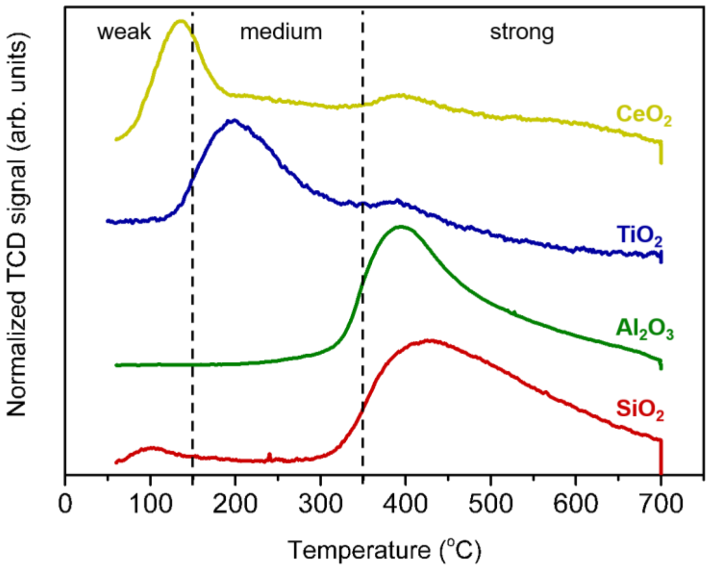

Supplementary Figure 10. CO_2-TPD profiles of the support materials used in this study recorded with a thermal conductivity detector (TCD).

Supplementary Table 4. Basicity of the support materials used in this study estimated from CO_2-TPD data.

**Sample**	**CO_2-TPD integral (arb. units)**	**Sample mass (g)**	**BET surface area ($m^2 g^{-1}$)**	**Basicity (μmol CO_2 m^{-2})**
SiO_2	43.3	0.0980	300	9.1
Al_2O_3	107	0.1090	262	15
TiO_2	85.7	0.0990	52.7	68
CeO_2	71.4	0.1004	88.5	33

2.5. Catalytic performance

Supplementary Table 5. Catalytic performance of the cobalt-based catalysts under study. T=250°C, P=20 bar, H_2/CO_2=3 GHSV=3000 h^{-1}, 10 h time-on-stream.

**Catalyst**	**CO_2 conversion (%)**	**CO selectivity (%)**	**CH_4 selectivity (%)**	**C_2 selectivity (%)**	**C_2 O/P[a]**	**C_3 selectivity (%)**	**C_3 O/P[a]**	**C_4 selectivity (%)**
Co/SiO_2-ox	1.13 ± 0.12	11.9 ± 3.7	86.1 ± 3.6	1.69 ± 0.11	0.003	0.34 ± 0.03	0.01	n.a.
Co/SiO_2-red	3.02 ± 0.12	5.38 ± 0.25	92.1 ± 0.2	2.03 ± 0.07	0.002	0.48 ± 0.02	0.01	n.a.
Co/Al_2O_3-ox	1.21 ± 0.11	0.00 ± 0.00	89.9 ± 19.6	6.60 ± 16.2	0.90	3.44 ± 10.4	1.54	n.a.
Co-Al_2O_3-red	1.35 ± 0.33	60.8 ± 0.8	38.8 ± 0.8	0.35 ± 1.54	0.66	0.05 ± 0.00	1.15	n.a.
Co/TiO_2-ox	5.46 ± 0.81	5.26 ± 4.12	85.6 ± 13.6	8.87 ± 8.90	0.40	1.72 ± 2.18	1.68	0.18 ± 0.87

Co/TiO_2-red	4.12 ± 0.96	1.24 ± 1.11	95.2 ± 7.94	2.19 ± 2.24	0.002	2.14 ± 1.44	0.01	0.11 ± 0.37
Co/CeO_2-ox	2.77 ± 0.25	4.38 ± 2.10	94.9 ± 2.1	0.66 ± 0.06	0.01	0.04 ± 0.02	0.13	n.a.
Co/CeO_2-red	3.98 ± 1.60	1.97 ± 0.64	97.5 ± 0.6	0.49 ± 0.01	0.01	0.05 ± 0.00	0.04	n.a.

[a] O/P stands for olefin/paraffin.

2.6. Density functional theory calculations

To gain a theoretical understanding of the differences in CO_2 adsorption on CoO versus metallic Co, we performed density functional theory (DFT) calculations on the face-centered cubic (FCC) CoO and metallic Co surface facets. We performed geometry optimizations of Co(110), Co(111), and CoO(100), the most active facets, both with and without adsorbed CO_2. The obtained structures are visualized in **Supplementary Figure 11**. The geometry optimization of CO_2 on the Co(111) surface resulted in a stable structure with a positive adsorption energy of 35.4 kJ/mol, indicating an endothermic process and it is likely that CO_2 activation does not take place on this facet[32]. The stable structure on the Co(110) surface led to a negative adsorption energy of -63.2 kJ/mol and -34.1 kJ/mol was obtained on CoO(100) (**Supplementary Table 6**). These results indicate that both Co(110) and CoO(100) can activate CO_2 molecules upon adsorption. Besides, we evaluated C-O bond elongation and the angle of the O-C-O bond after adsorption to qualitatively assess to what extend CO_2 is activated upon adsorption on the three surfaces. The sp hybridized free CO_2 molecule possesses a O-C-O bond angle of 180°. To activate a CO_2 molecule on a catalytic surface, the angle of O-C-O has to change. From the results in **Supplementary Table 6**, we can learn for example that CO_2 is least deformed, to 145.4°, on CoO(100) after adsorption, as the O-C-O bond angle changes the least. The highest deformation of 120.6° was observed on Co(110). Besides, C-O bond elongation and thus potentially dissociation is most severe on Co(110). These theoretical results indicate that metallic cobalt activates CO_2 molecules more compared to CoO, which is in line with our experimental observations, where we observe CO_{ads} only on metallic cobalt. Please note that we have adopted a simplified approach herein, reporting the optimized geometry. There are other possibilities in which CO_2 can bind to each of the chosen surfaces, depending on the orientation of the CO_2 adsorbate with respect to the surface as well as the chosen adsorption site of the surface. This generally results in varying possibilities for the adsorption energy[32]. For example, oxygen vacancies on the cobalt oxide surface could be good docking sites for CO_2, which would facilitate the adsorption[33]. Moreover, the support material, which we excluded from our calculations, plays a significant role in the resulting adsorption energy[34].

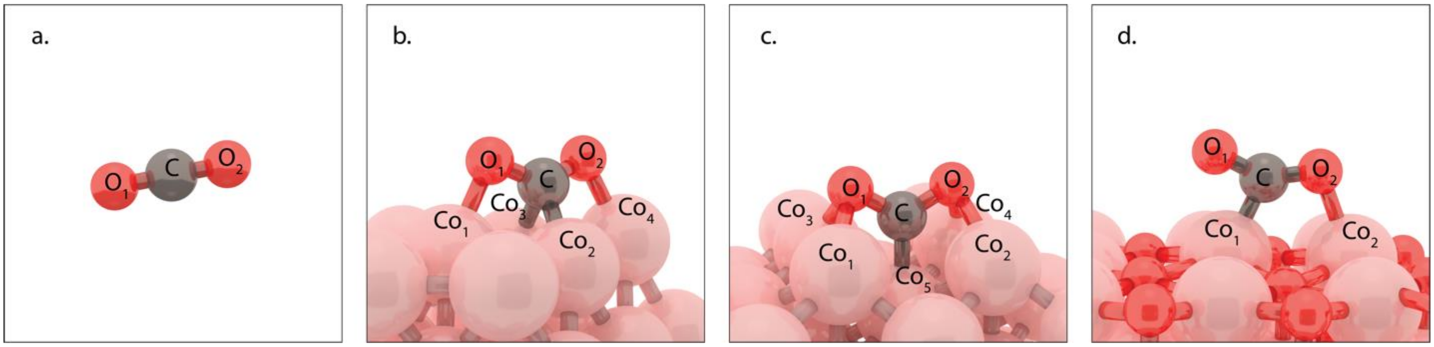

Supplementary Figure 11. Visualizations of geometry optimization using density functional theory (DFT). **a** A free CO_2 molecule. CO_2 adsorbed on **b** Co(111) **c** Co(110) and **d** CoO(100).

Supplementary Table 6. Adsorption energies and structural parameters of CO_2 adsorbed on Co(110), Co(111), and CoO(100).

	**CO_2 (g)**	***CO_2 on Co(110)**	***CO_2 on Co(111)**	***CO_2 on CoO(100)**
E_{ads} (kJ/mol)		-63.2	35.4	-34.1
O—C—O (°)	180	120.6	129.5	145.4
C—O_1 (Å)	1.18	1.32	1.28	1.23
C—O_2 (Å)	1.18	1.31	1.28	1.24
O_1—Co_1 (Å)		2.08	2.08	
O_2—Co_4 (Å)		2.08	2.08	
O_1—Co_3 (Å)		2.08		
O_2—Co_2 (Å)		2.09		1.12
C—Co_1 (Å)				1.93
C—Co_2 (Å)			1.93	
C—Co_3 (Å)			2.21	
C—Co_5 (Å)		1.90		

2.7. Modulation excitation diffuse reflectance infrared Fourier transform spectroscopy

Phase-resolved DRIFTS data

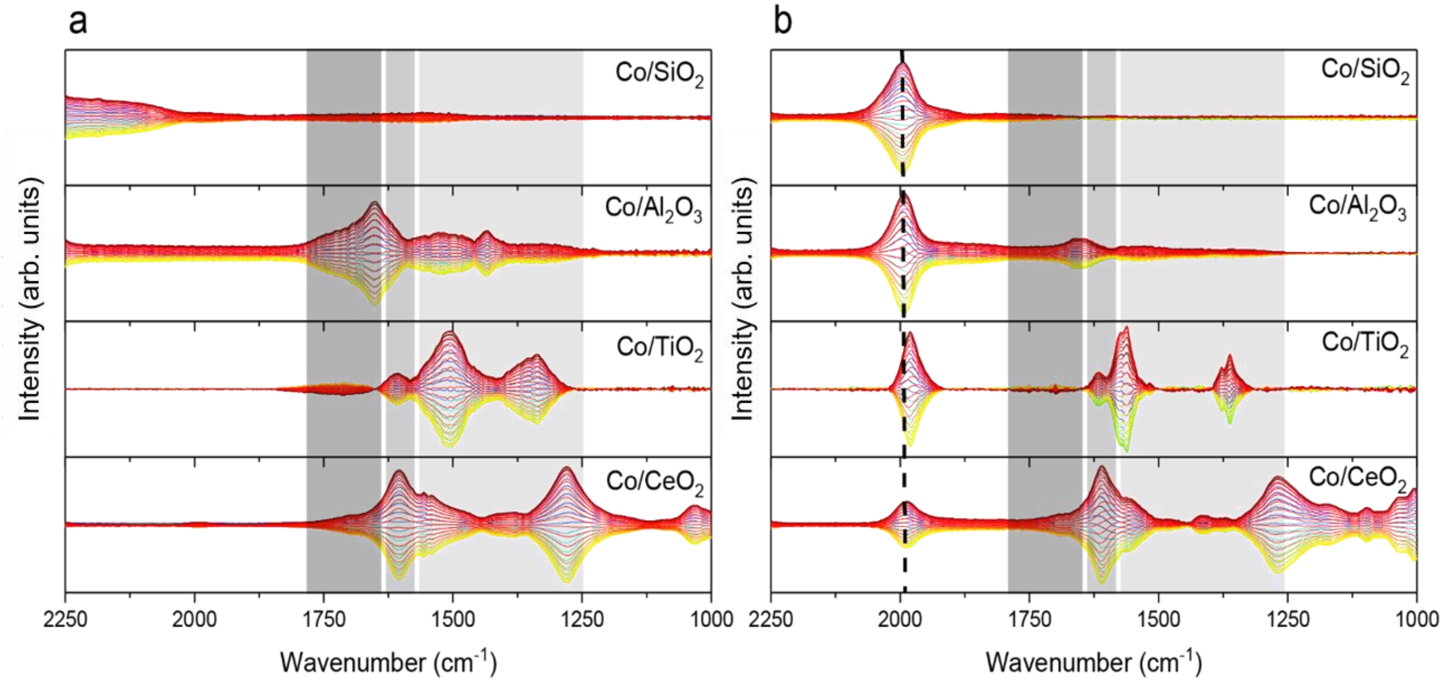

Supplementary Figure 12. Phase-resolved DRIFT spectra of the supported cobalt catalysts (T=250°C, H_2/CO_2=3, P=1 bar) in **a** CoO and **b** metallic Co state. Spectra are within φ^{PSD}= 0-360° at steps of φ^{PSD}= 10°. Adsorbed surface species (see also **Figure 3** in the main text) are indicated as follows: CO (black dashed line), formyl (dark gray panel), formate (medium gray panel), (bi)carbonates (light gray panel).

Peak assignments

All peak assignments for the phase-resolved amplitude spectra in **Figure 2** in the main text and **Supplementary Figure 12** can be found in **Supplementary Table 7**. The peaks for bidentate carbonate species (indicated in green in **Supplementary Table 7**) have been used to assess the basicity of O atoms for Co/Al_2O_3, Co/TiO_2, and Co/CeO_2 (**Supplementary Table 8**). A higher value of $\Delta v_{as\text{-}s}$ (*i.e.* the difference between the asymmetric and the symmetric stretching vibration) means less basic O atoms and thus less reactivity[35]. Co/Al_2O_3 displayed the highest $\Delta v_{as\text{-}s}$ values both in CoO and metallic Co state, indicating that the bidentate carbonate species had the lowest reactivity. The catalysts with reducible supports, TiO_2 and CeO_2, exhibited lower values for $\Delta v_{as\text{-}s}$ and their bidentate carbonate species were thus more reactive compared to the species on Co/Al_2O_3. The lowest $\Delta v_{as\text{-}s}$ value was observed for Co/TiO_2-ox, which was in line with the best catalytic performance displayed by this catalyst.

Supplementary Table 7. Peak assignments in the phase-resolved amplitude spectra in **Figure 2** in the main text. The values indicated in green have been used to assess basicity of O atoms by calculating $\Delta v_{as\text{-}s}$(OCO) for bidentate carbonate species in **Supplementary Table 8**.

**CoO**			**Metallic Co**		
**Catalyst**	**IR band (cm^{-1})**	**Assignment**	**Catalyst**	**IR band (cm^{-1})**	**Assignment**
Co/SiO_2	2360	CO_2 (gas)[36]	Co/SiO_2	2358	CO_2 (gas)[36]
	2344	CO_2 adsorbed (linear)[37]		2344	CO_2 adsorbed (linear)[37]
	2185	CO (gas)[38]		1994	CO (bridged/multi-bonded)[39]

				1816	CO (3-fold coordinated)[40]
Co/Al_2O_3	2358	CO_2 (gas)[36]	Co/Al_2O_3	2358	CO_2 (gas)[36]
	2344	CO_2 adsorbed (linear)[37]		2344	CO_2 adsorbed (linear)[37]
	1726	ν(C=O) CHO (formyl) [35,41,42]		2186	CO (gas)[38]
	1654	HCO_2^{2-} (m-formate)[38]		1992	CO (bridged/multi-bonded)[39]
	1558	$\nu_{sym}(CO_3^{2-})$ (bidentate)[38,40]		1846	CO (3-fold coordinated)[40]
	1522	$\nu_{sym}(CO_3^{2-})$ (bidentate)[38,40]		1664	HCO_2^{2-} (m-formate)[38]
	1485	$\nu_{asym}(CO_3^{2-})$ (m-/p-dentate)[38]		1573	$\nu_{sym}(CO_3^{2-})$ (bidentate)[38,40]
	1435	$\nu_{sym}(CO_3^{2-})$ (m-/p-dentate)[38]		1358	$\nu_{asym}(CO_3^{2-})$ (bidentate)[38,40]
	1355	$\nu_{asym}(CO_3^{2-})$ (bidentate)[38,40]			
	1328	$\nu_{asym}(CO_3^{2-})$ (bidentate)[38,40]			
Co/TiO_2	2360	CO_2 (gas)[36]	Co/TiO_2	2360	CO_2 (gas)[36]
	2343	CO_2 (adsorbed)		2343	CO_2 (adsorbed)
	1709	ν(C=O) CHO (formyl)[35,41,42]		1980	CO (bridged/multi-bonded)[39]
	1606	HCO_2^{2-} (b-formate)[37,38,40]		1706	ν(C=O) CHO (formyl)[35,41,42]
	1508	$\nu_{sym}(CO_3^{2-})$ (bidentate)[38,40]		1615	HCO_2^{2-} (b-formate)[37,38,40]
	1460	δ(C-H_2) scissor[43–45]		1574,	$\nu_{sym}(CO_3^{2-})$ (bidentate)[38,40]
	1339	$\nu_{asym}(CO_3^{2-})$ (bidentate)[38,40]		1562	$\nu_{asym}(CO_3^{2-})$ (bidentate)[38,40]
				1378,	
				1362	
Co/CeO_2	2358	CO_2 (gas)[36]	Co/CeO_2	2358	CO_2 (gas)[36]
	2343	CO_2 (asorbed)		2343	CO_2 (adsorbed)
	1692	ν(C=O) CHO (formyl)[35,41,42]		1987	CO (bridged/multi-bonded)[39]
	1603	HCO_2^{2-} (b-formate)[37,40]		1690	ν(C=O) CHO (formyl)[35,41,42]
	1556	$\nu_{sym}(CO_3^{2-})$ (bidentate)[38,40,46]		1610	HCO_2^{2-} (b-formate)[37,40]
	1542	HCO_2^{2-} (br-formate)[47]		1562	$\nu_{sym}(CO_3^{2-})$ (bidentate)[38,40,46]
	1409	CO_3^{2-} (monodentate)[48]		1413	$\nu_{sym}(HCO_3^{-})$ [38,40]
	1380	$\nu_{asym}(CO_3^{2-})$ (bidentate)[38,40]		1462	$\nu_{asym}(HCO_3^{-})$ [38,40,46]
	1281	ν(C-O) HCO_2^{2-} (m-formate)[35,38]		1370	$\nu_{asym}(CO_3^{2-})$ (bidentate)[38,40]
	1033	HCO_3^{-} [46,47]		1269	ν(C-O) HCO_2^{2-} (m-formate)[35,38]
				1562,	HCO_2^{2-} (br-formate)[38]
				1370	
				1174	CO_3^{2-} (br-carbonate)[47]
				1098	HCO_3^{-} [46,47]

ν: stretching vibration
δ: bending vibration
ν_{sym}: symmetric stretching vibration
ν_{asym}: asymmetric stretching vibration
Formates (HCO_2^{2-}): m=monodentate, b=bidentate, br=bridged
Carbonates (CO_3^{2-}): m=monodentate, b=bidentate, p=polydentate
Bicarbonates (HCO_3^{-})

Supplementary Table 8. $\Delta\nu_{as-s}$(OCO) for bidentate carbonate species on the cobalt-based catalysts. The peaks used for this are indicated in green in **Supplementary Table 7**.

**CoO**		**Metallic Co**	
**Catalyst**	**$\Delta\nu_{as-s}$(OCO)[35]**	**Catalyst**	**$\Delta\nu_{as-s}$(OCO)[35]**

Co/SiO_2	N.A.	Co/SiO_2	N.A.
Co/Al_2O_3	197	Co/Al_2O_3	215
Co/TiO_2	169	Co/TiO_2	200, 204
Co/CeO_2	176	Co/CeO_2	192

* Higher value of $\Delta v_{as\text{-}s}$ means less basic O atoms and less reactive[35]

Mass spectrometry signals during ME DRIFTS

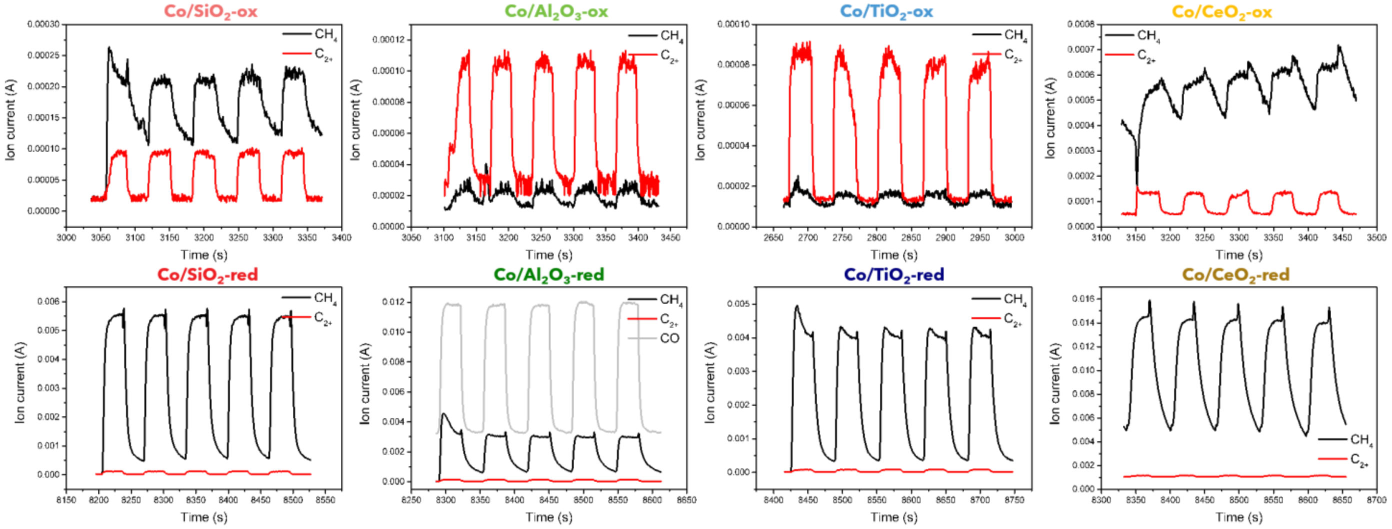

Supplementary Figure 13. Mass spectrometry signals of CH_4 (black, m/z=15) and C_{2+} hydrocarbons (red, m/z=29) during the ME DRIFTS experiments on (from left to right) Co/SiO_2, Co/Al_2O_3, Co/TiO_2, and Co/CeO_2. The top panel displays the CoO-containing (suffix: -ox) catalysts and the bottom panel the metallic Co (suffix: -red) catalysts. For Co/Al_2O_3-red the CO signal (gray, m/z=28) was significant and added as well.

Kinetics from phase delays

The phase-resolved amplitude spectra and corresponding phase shifts for the set of cobalt-based catalysts can be found in **Supplementary Figure 14a** and **b**, respectively. The phase shifts for selected species (adsorbed CO, (bi)carbonates, and formates) can be found in **Supplementary Figures 15** and **16**. The slopes of the desorption branches taken from the first 10 s after turning the CO_2 gas off can be found for the same selected species in **Figure 4** in the main text and **Supplementary Figure 17**. The slopes of the desorption branches were determined by assuming a straight line based on 10 points (**Supplementary Figure 18**). The R^2 values for the straight lines were between 0.73 and 0.97 for all species.

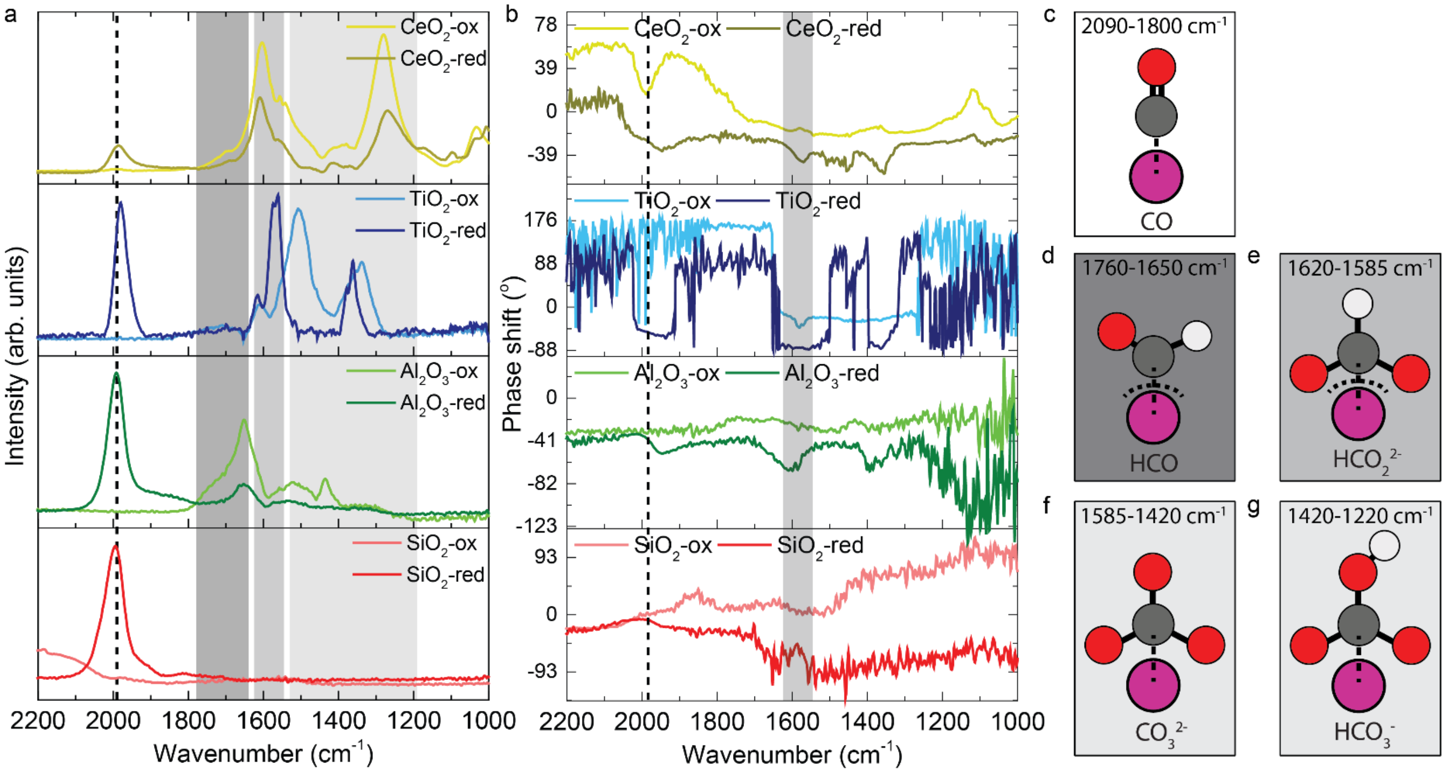

Supplementary Figure 14. Modulation excitation infrared spectroscopy (ME DRIFTS) results for the cobalt-based catalysts. a Phase-resolved amplitude spectra and **b** phase shifts/delays for CoO (suffix: -ox) and metallic Co (suffix: -red) supported catalysts (T=250°C, P=1 bar, H_2/CO_2=3). **c-g** Adsorbed surface species with typical vibration energies: **c** CO, **d** formyl, **e** formate, **f** carbonate, and **g** bicarbonate. Data in **a** are absolute values of the spectra, meaning all signals appear positive for the sake of clarity.

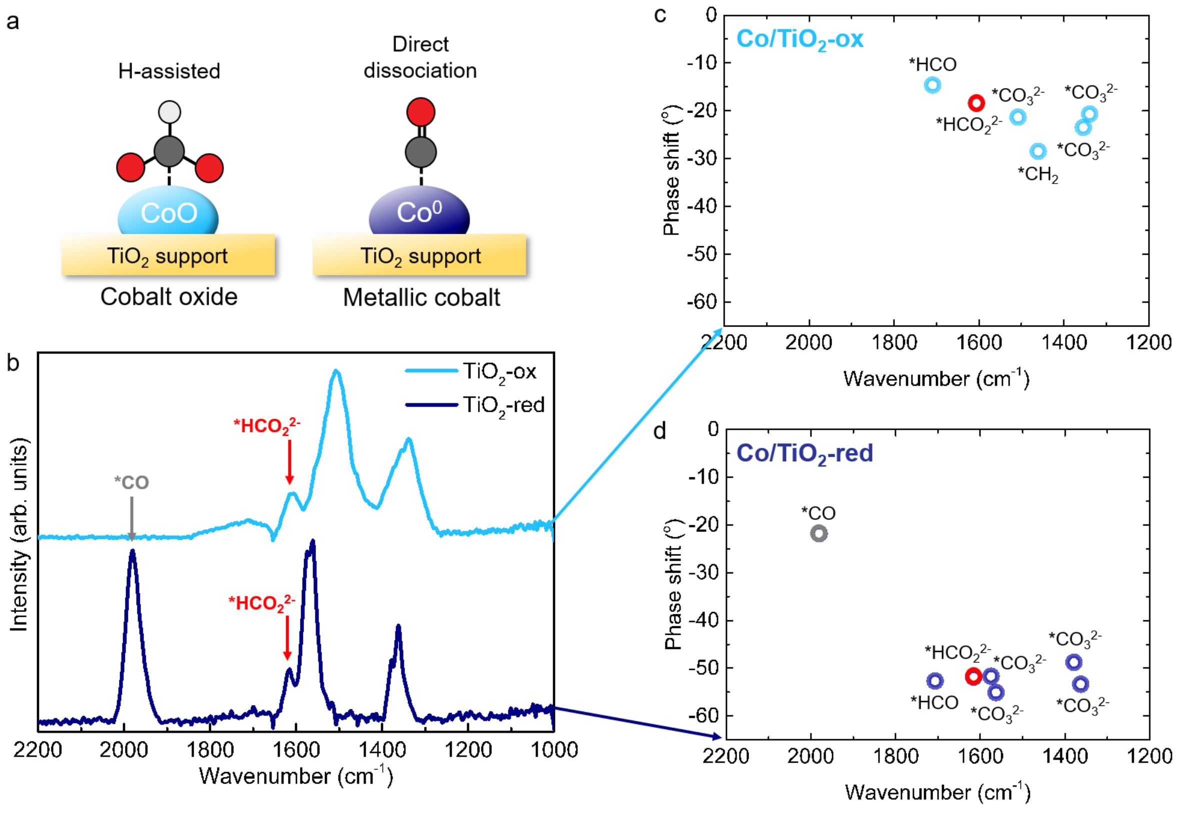

Supplementary Figure 15. Kinetic information derived from the phase shift. a Schematic representation of the H-assisted mechanism, dominant for CoO (suffix: -ox) catalysts, and the direct dissociation mechanism, dominant for metallic Co (suffix: -red) catalysts. **b** Phase-resolved amplitude spectra for Co/TiO_2-ox and Co/TiO_2-red. Phase shifts of selected species from the phase-resolved amplitude spectra for **c** Co/TiO_2-ox and **d** Co/TiO_2-red. Both samples showed carbonate, formate (*HCO_2^{2-} indicated in red; 1609-1615 cm^{-1}), and formyl species. Co/TiO_2-ox additionally showed *CH_2 species. For Co/TiO_2-red, *CO (gray; 1980 cm^{-1}) displayed faster kinetics (smaller phase shift) than the carbonate, formate, and formyl species. The direct dissociation mechanism was thus faster than the H-assisted mechanism.

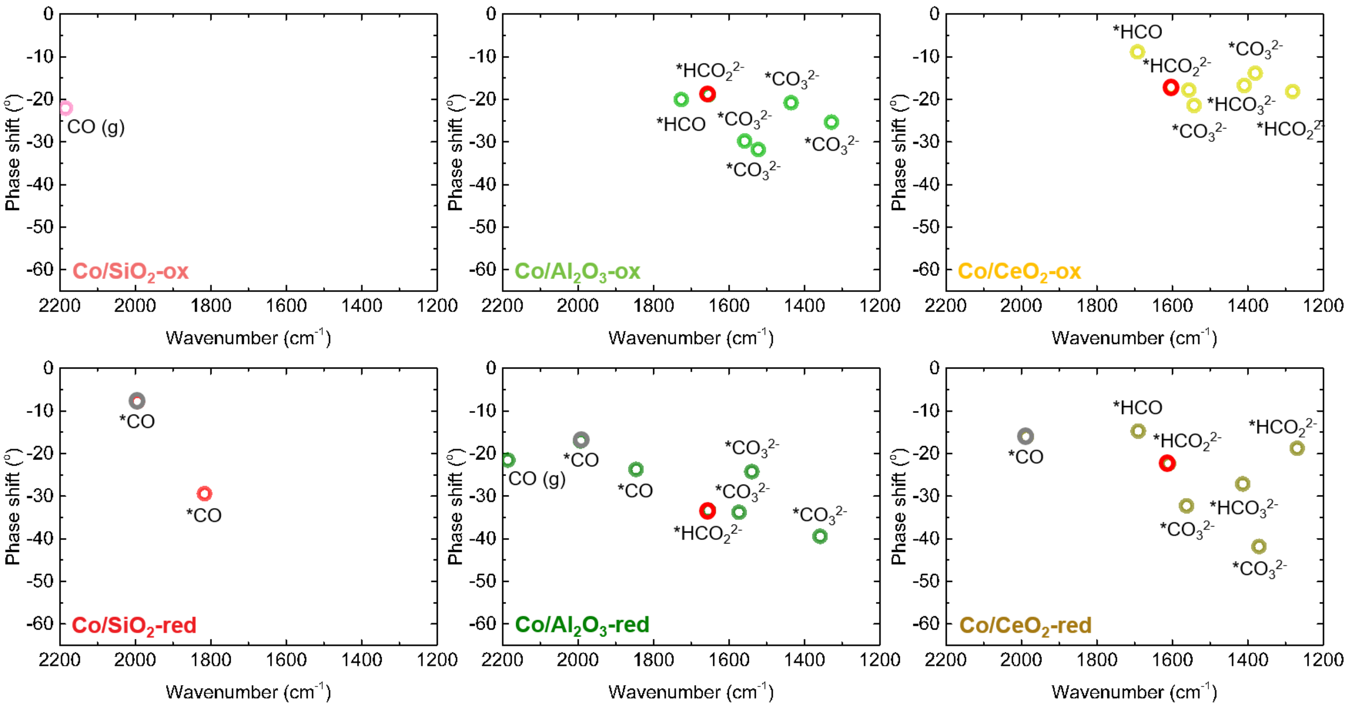

Supplementary Figure 16. Phase shifts for (from left to right) Co/SiO_2, Co/Al_2O_3, and Co/CeO_2, derived from the phase-resolved amplitude spectra. The top panel displays the CoO-containing (suffix: -ox) catalysts and the bottom panel the metallic Co (suffix: -red) catalysts. Co/SiO_2-ox only showed gaseous CO, while Co/SiO_2-red only displayed *CO. Co/Al_2O_3-ox and Co/CeO_2-ox showed carbonate, formate ($*HCO_2^{2-}$ indicated in red; 1609-1615 cm^{-1}), and formyl species. The metallic Co catalysts (bottom) displayed *CO (gray; 1980-1994 cm^{-1}), which had mostly faster kinetics (smaller phase shift) compared to the carbonate, formate, and formyl species.

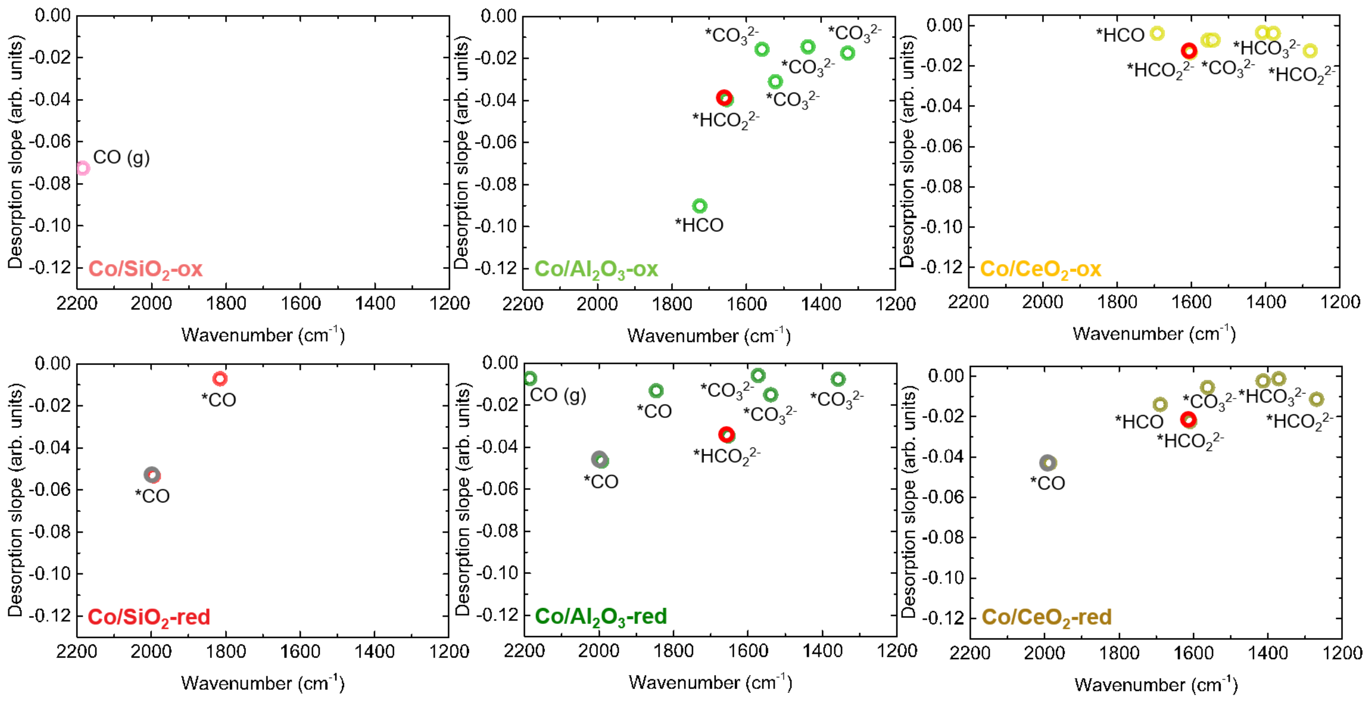

Supplementary Figure 17. Slopes of the desorption branches of the time-resolved DRIFT spectra after turning off the CO_2 gas for (from left to right) Co/SiO_2, Co/Al_2O_3, and Co/CeO_2. The top panel displays the CoO-

containing (suffix: -ox) catalysts and the bottom panel the metallic Co (suffix: -red) catalysts. Co/SiO_2-ox only showed gaseous CO, while Co/SiO_2-red only displayed *CO. Co/Al_2O_3-ox and Co/CeO_2-ox showed carbonate, formate (*HCO_2^{2-} indicated in red; 1609-1615 cm^{-1}), and formyl species. The metallic Co catalysts (bottom) displayed *CO (gray; 1980-1994 cm^{-1}), which had faster kinetics (steeper slopes) compared to the carbonate, formate, and formyl species.

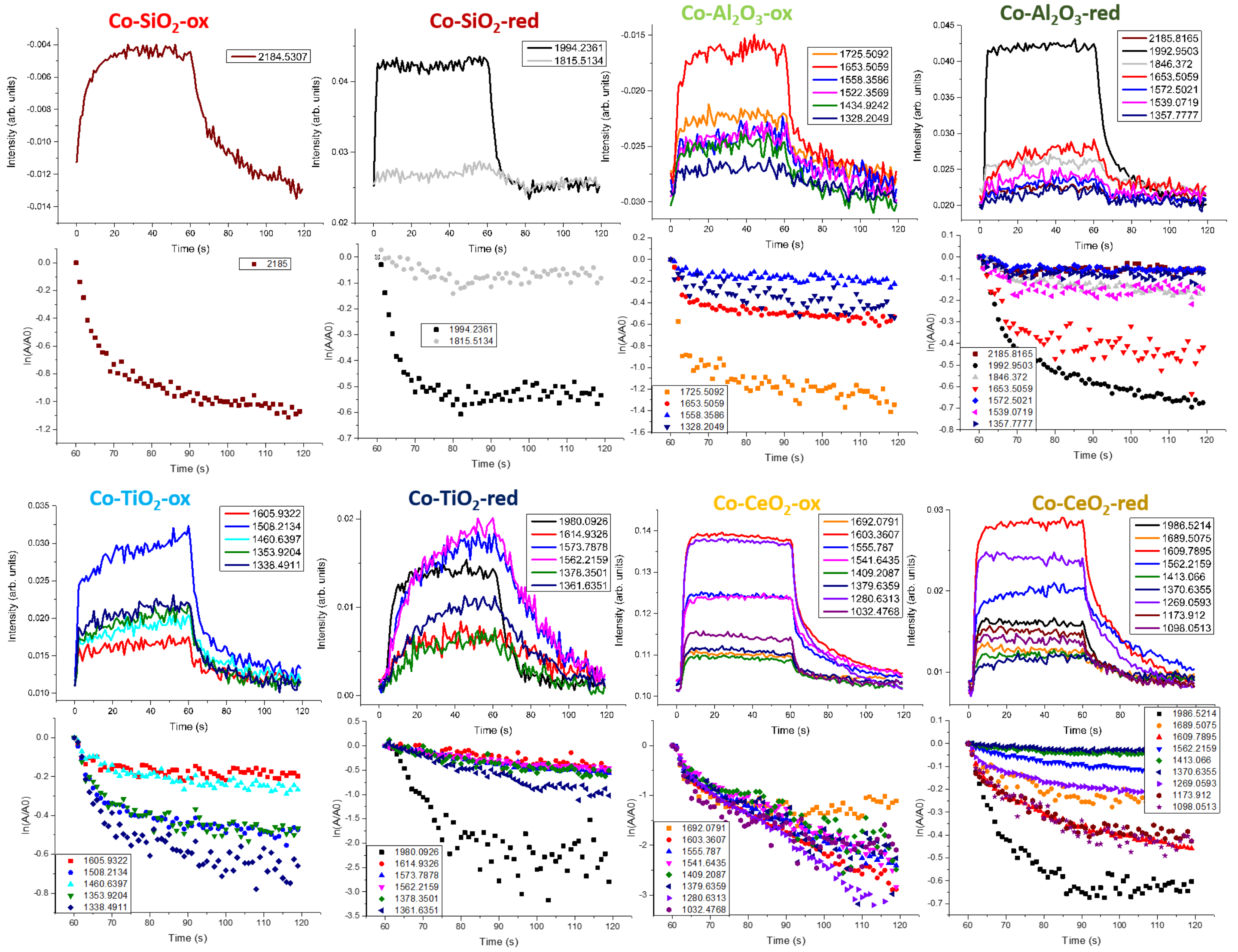

Supplementary Figure 18. Determination of the slopes of the desorption profiles of selected species in the time resolved DRIFT spectra. In the row 1 and 3, the temporal dependence of the intensity of the selected species is plotted, while that of the corresponding ln(A/A_0) values is shown in row 2 and 4. A is the intensity of the species and A_0 is the intensity of the species at t=60 s (*i.e.* when the CO_2 is turned off). Suffix -ox denotes the CoO catalysts and suffix -red denotes the metallic Co catalysts.

2.8. Co-feeding CO during CO_2 hydrogenation and comparison of catalytic performance

Supplementary Table 9. Catalytic performance of the cobalt-based catalysts under study. T=250°C, P=20 bar, H_2/CO_2=3, GHSV=3000 h^{-1}, 10 h time-on-stream. For co-feeding CO_2/CO=2. Suffix -ox denotes the CoO catalysts (pre-treated at 250°C in N_2/H_2=2) and suffix -red denotes the metallic Co catalysts (pre-treated at 450°C in N_2/H_2=2).

Catalyst	CO_2 conversion (%)	CO selectivity or conversion (%)	CH_4 selectivity (%)	C_2 selectivity (%)	C_2 O/P[b]	C_3 selectivity (%)	C_3 O/P[b]	C_4 selectivity (%)	C_4 O/P[b]	C_{5+} selectivity (%)
Co/TiO2-ox	5.46 ± 0.81	5.26 ± 4.12	85.6 ± 13.6	8.87 ± 8.90	0.40	1.72 ± 2.18	1.68	0.18 ± 0.87	0.11	n.a.
Co/TiO2-ox co-feed	14.9[a] ± 3.24	22.7 ± 1.71	61.7 ± 2.78	11.1 ± 2.58	0.90	10.8 ± 1.73	4.53	9.87 ± 0.95	5.54	5.37 ± 2.21
Co/TiO2-ox FTS	n.a.	13.8 ± 0.41	53.8 ± 2.63	6.72 ± 0.30	9.32	12.9 ± 0.67	10.7	13.8 ± 1.05	8.35	12.6 ± 1.29
Co/TiO2-red	4.12 ± 0.96	1.24 ± 1.11	95.2 ± 7.94	2.19 ± 2.24	0.002	2.14 ± 1.44	0.01	0.11 ± 0.37	P	n.a.
Co/TiO2-red co-feed	14.1[a] ± 0.73	28.0 ± 0.47	87.0 ± 1.21	6.97 ± 0.39	O	3.87 ± 0.47	1.97	1.69 ± 0.31	1.47	0.43 ± 0.08
Co/TiO2-red FTS	n.a.	12.6 ± 0.27	54.0 ± 4.45	9.81 ± 2.00	0.35	16.8 ± 3.35	3.63	13.8 ± 3.52	2.22	5.48 ± 2.50

[a] The total amount of CO_2 during co-feeding CO (4 mL min^{-1}) was lower than during CO_2 hydrogenation only (8 mL min^{-1} *vs.* 12 mL min^{-1}). For example, the absolute CO_2 conversion for Co/TiO_2-ox is (5.46%*12 mL min^{-1}=) 0.65 mL min^{-1}, while Co/TiO_2-ox co-feed converts (14.9%*8 mL min^{-1}=) 1.20 mL min^{-1} CO_2.
[b] O/P stands for olefin/paraffin. O means olefin only and P means paraffin only.

2.9. Kinetic parameters for Co-TiO_2-ox and Co/TiO_2-red

For the best performing catalyst in our study, Co-TiO_2, we additionally determined a set of kinetic parameters at P=20 bar in both the CoO and metallic Co state. The apparent activation energy (Ea) for CO_2 hydrogenation was slightly lower for Co/TiO_2-ox, 113 ± 3, compared to Co/TiO_2-red, 122 ± 5 (**Supplementary Figures 19, 20** and **Table 1** in the main text). This is in line with the better performance of Co/TiO_2-ox compared to Co/TiO_2-red. To gain more insights, we also determined the reaction orders in CO_2 and in H_2 for both samples. The higher reaction order in CO_2 of Co/TiO_2-ox (0.38 ± 0.09) compared to Co/TiO_2-red (0.15 ± 0.04) indicated that a strongly adsorbed intermediate derived from CO_2 on the Co/TiO_2-red surface, most likely adsorbed CO, hinders the reaction[47]. For metallic Co, a reaction order of 0.14 in CO_2 has been reported previously for the CO_2 hydrogenation reaction[49]. Besides, the reaction orders in H_2 were almost completely opposite for the 2 samples: a positive order of 1.24 ± 0.40 for Co/TiO_2-ox versus a negative order of -1.15 ± 0.07 for Co/TiO_2-red. This particularly substantiates the hypothesis that the Co/TiO_2-ox catalyst, following the H-assisted mechanism, benefits from a higher partial pressure in H_2. On the other hand, the Co/TiO_2-red catalyst, following mainly the direct dissociation mechanism, benefits from a lower partial pressure in H_2, as H_2 may be competing with adsorbed CO, the most important intermediate in the direct dissociation mechanism.

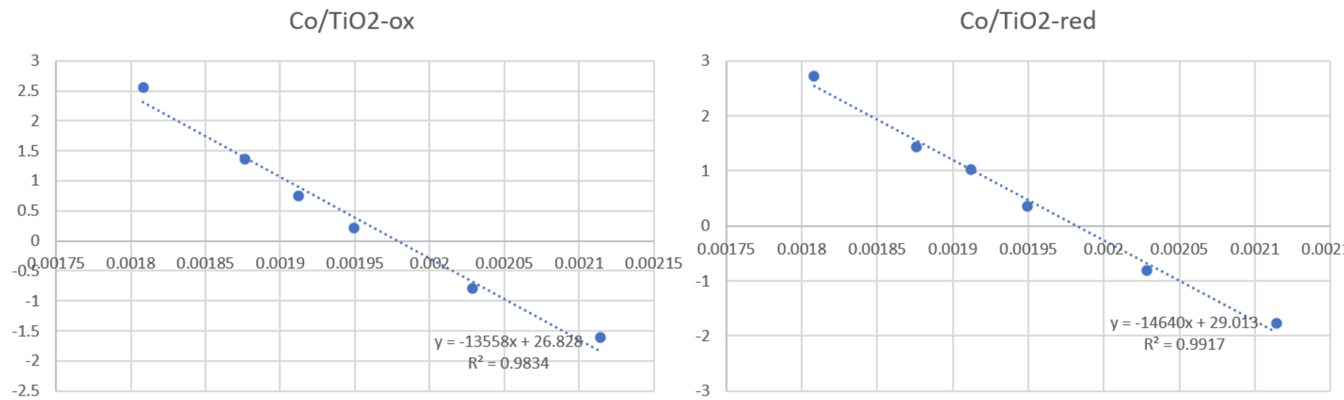

Supplementary Figure 19. Arrhenius plots for Co/TiO_2-ox (left, pre-treated at 250°C in N_2/H_2=2) and Co/TiO_2-red (right, pre-treated at 450°C in N_2/H_2=2). The activities were measured between 200 and 280°C after 1 h of stabilization with at least 4 GC injection points at each temperature point.

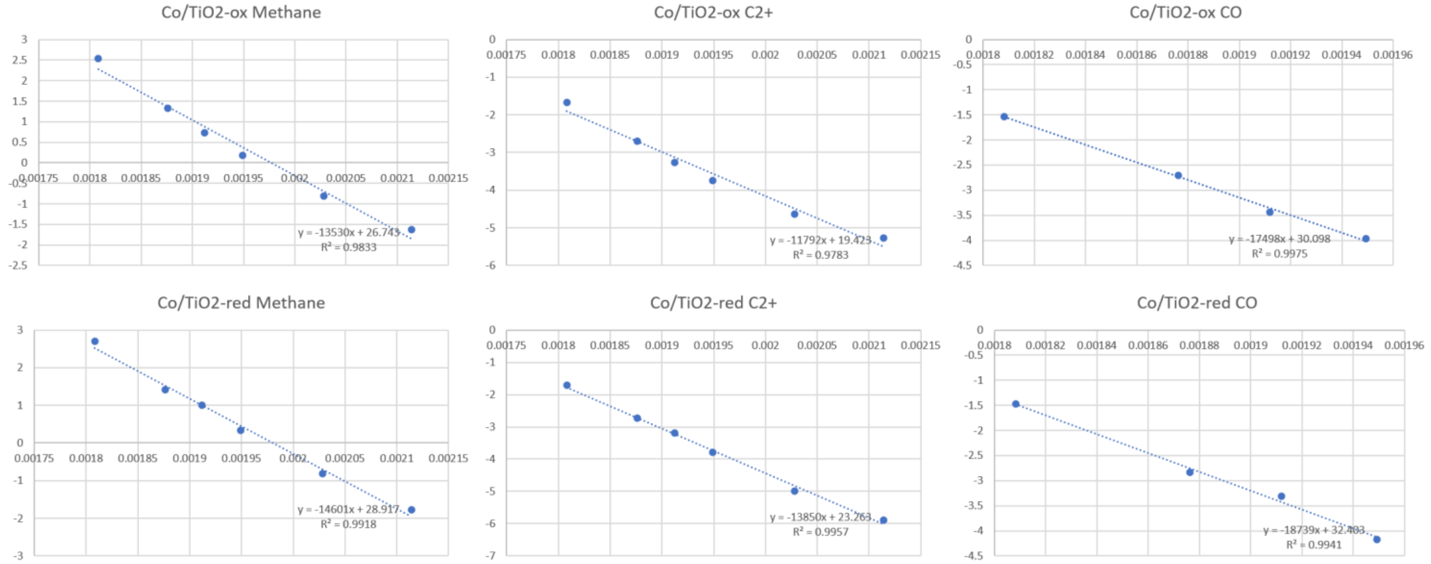

Supplementary Figure 20. Arrhenius plots for selected products CH_4, C_{2+}, and CO for Co/TiO_2-ox (top, pre-treated at 250°C in N_2/H_2=2) and Co/TiO_2-red (bottom, pre-treated at 450°C in N_2/H_2=2). The activities were measured between 200 and 280°C after 1 h of stabilization with at least 4 GC injection points at each temperature point. Note that CO was not produced below 240°C.

2.10. Thermodynamics

The thermodynamic feasibility of the different cobalt phases metallic Co, CoO, Co_3O_4, and Co_2C was assessed with thermodynamic calculations. During CO_2 hydrogenation, co-feeding CO_2 and CO, and FTS (**Supplementary Figure 21a-c**), metallic Co and CoO were both thermodynamically feasible. Co_3O_4 and Co_2C, however, were not present in significant amounts during the simulation at 250°C while varying the pressure between 1 and 20 bar. Additionally, under FTS conditions we varied the H_2/CO ratio between 0 and 4 at constant temperature (250°C) and pressure (20 bar)(**Supplementary Figure 21d**). We found that the formation of Co_2C was only thermodynamically feasible at $H_2/CO<1$. Though, at typical cobalt-based FTS conditions (H_2/CO=2) metallic Co and CoO were the dominant phases. Co_3O_4 was again not significantly present during the simulation.

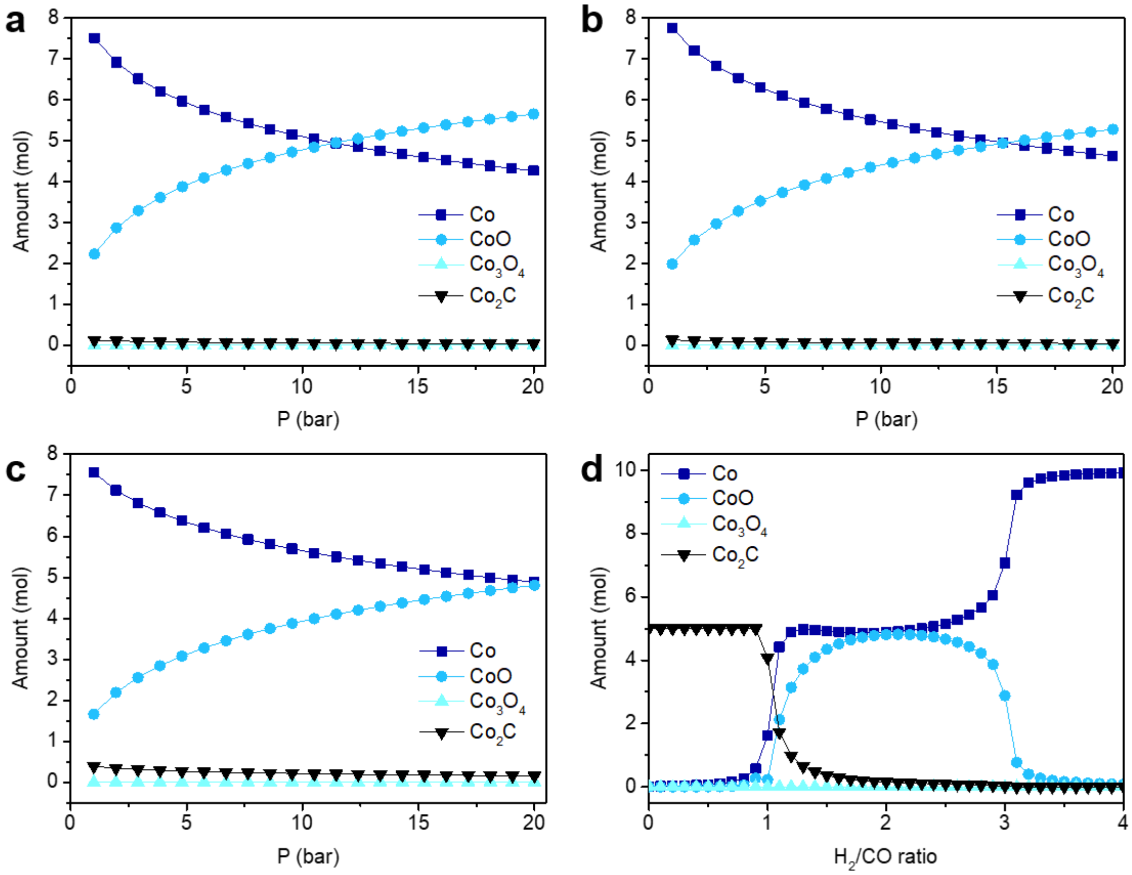

Supplementary Figure 21. **Thermodynamic stability of different cobalt phases under relevant reaction conditions. a** CO_2 hydrogenation at *T*=250°C and H_2/CO_2=3 while varying the pressure. **b** *T*=250°C and $H_2/CO_2/CO$=9/2/1 while varying the pressure. **c** Fischer-Tropsch Synthesis (FTS) at *T*=250°C and H_2/CO=2 while varying the pressure. **d**, Varying the H_2/CO ratio during FTS at *T*=250°C and *P*=20 bar.

2.11. Long-term stability testing for Co/TiO_2

Long-term stability was tested for Co/TiO_2-ox (**Figure 5cd** in the main text) and Co/TiO_2-red (**Supplementary Figure 22**). The tests were performed at 250°C and 20 bar for 150 h in total: first for 50 h under CO/CO_2 co-feeding conditions (CO_2/CO=2) and then for 100 h under CO_2 hydrogenation conditions (H_2/CO_2=3). For Co/TiO_2-ox (**Figure 5cd** in the main text), during the 50 h of co-feeding, the total carbon conversion started at ~18% and stabilized after about 10 h to ~16%, while the C_{2+} selectivity started at ~40% and stabilized at ~35%. For the following 100 h of CO_2 conversion only, the conversion started at ~7.0% and remained ~4.5% after 100 h, while the C_{2+} selectivity increased from ~10% in the first few h to ~20% after 100 h, indicating that the activity loss over time was mostly related to a decrease in methane production. For Co/TiO_2-red (**Supplementary Figure 22**), during the 50 h of co-feeding, the total carbon conversion started at ~18% and decreased to ~16% after 50 h, while the C_{2+} selectivity started at ~10% and increased to ~13%. For the following 100 h of CO_2 conversion only, the conversion remained stable at ~5.0% during the 100 h, while the C_{2+} selectivity remained stable around ~5.0% during the 100 h. After 150 h time-on-stream, we verified with XRD that Co/TiO_2-ox contained CoO and Co/TiO_2-red contained metallic cobalt (FCC) (**Supplementary Figure 23**).

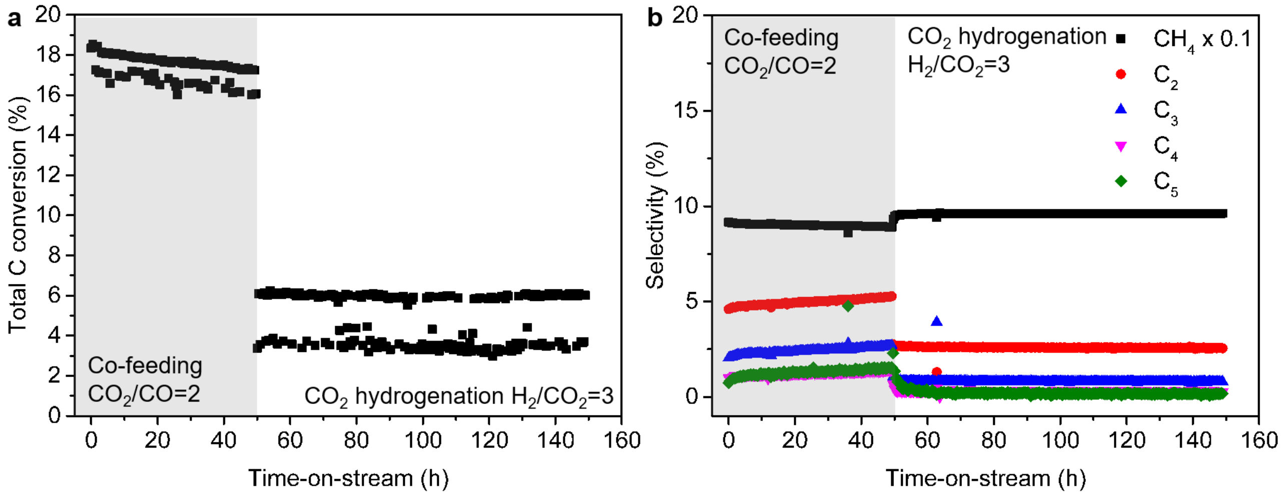

Supplementary Figure 22. Long-term stability test of Co-TiO_2-red. The reactor was operated for 150 h (T=250°C, P=20 bar, GHSV=3000 h^{-1}) **a** is displaying conversion and **b** selectivity first while co-feeding CO/CO_2 (H_2/CO_2/CO=9/2/1) for 50 h and then during CO_2 hydrogenation only (H_2/CO_2=3) for 100 h. The sample was treated at 450°C in N_2/H_2=2 prior to the reaction.

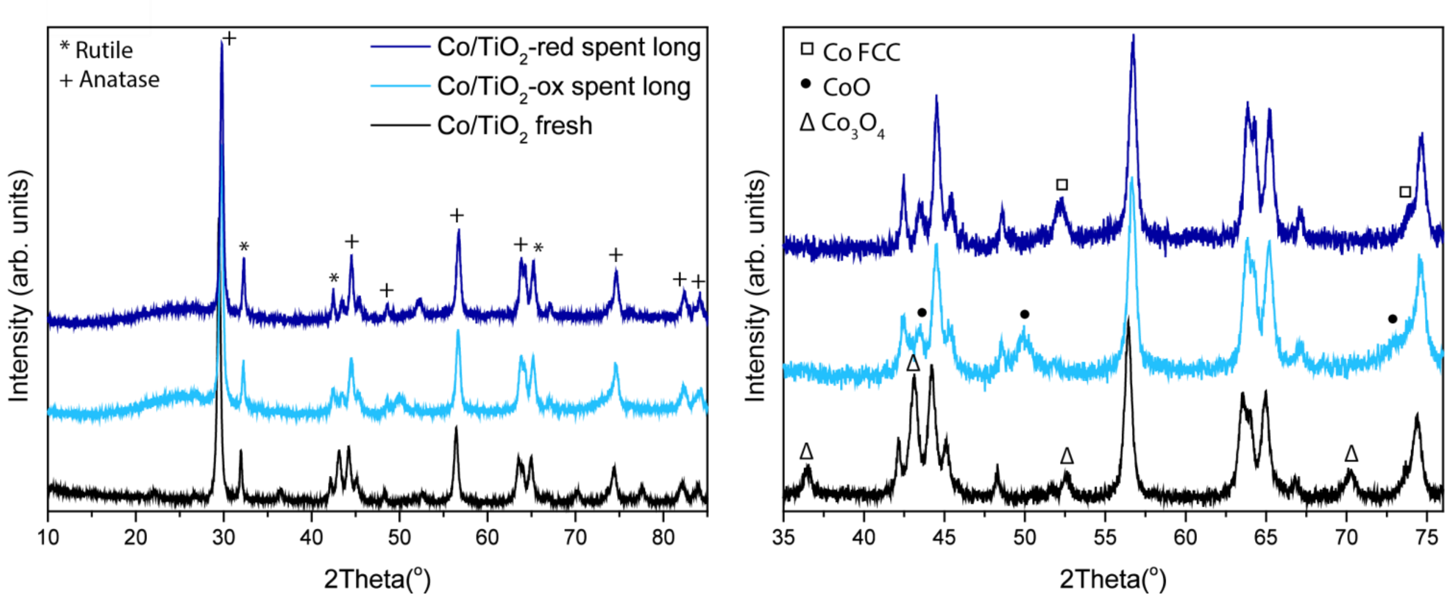

Supplementary Figure 23. X-ray diffraction of Co/TiO_2 after 150 h of catalytic testing. Diffraction patterns (left) of Co/TiO_2 fresh (black), Co/TiO_2-ox spent (pre-treated at 250°C in N_2/H_2=2), and Co/TiO_2-red spent (pre-treated at 450°C in N_2/H_2=2). The diffraction lines of rutile and anatase are indicated with stars and plusses. The zoom in (right) indicated Co_3O_4 (triangles) in the fresh catalyst, while Co/TiO_2-ox spent contained CoO (circles) and Co/TiO_2-red spent face-centered cubic (FCC) metallic cobalt (squares). Note that the spent catalysts were passivated in 1 vol.% O_2/Ar for 2 h at room temperature before they were taken out of the reactor.

Supplementary References

1. Blöchl, P. E. Projector augmented-wave method. *Phys. Rev. B* **50**, 17953–17979 (1994).

2. Joubert, D. From ultrasoft pseudopotentials to the projector augmented-wave method. *Phys. Rev. B - Condens. Matter Mater. Phys.* **59**, 1758–1775 (1999).

3. Perdew, J. P., Burke, K. & Ernzerhof, M. Generalized gradient approximation made simple. *Phys. Rev. Lett.* **77**, 3865–3868 (1996).

4. Kresse, G. & Furthmüller, J. Efficient iterative schemes for ab initio total-energy calculations using a plane-wave basis set. *Phys. Rev. B - Condens. Matter Mater. Phys.* **54**, 11169–11186 (1996).

5. Kresse, G. & Hafner, J. Ab initio molecular-dynamics simulation of the liquid-metal–amorphous-semiconductor transition in germanium. *Phys. Rev. B* **49**, 14251–14269 (1994).

6. Häglund, J., Fernndez Guillermet, A., Grimvall, G. & Körling, M. Theory of bonding in transition-metal carbides and nitrides. *Phys. Rev. B* **48**, 11685–11691 (1993).

7. Jauch, W., Reehuis, M., Bleif, H. J., Kubanek, F. & Pattison, P. Crystallographic symmetry and magnetic structure of CoO. *Phys. Rev. B - Condens. Matter Mater. Phys.* **64**, 2–4 (2001).

8. Monkhorst, H. J. M. & Pack, J. D. Special points for Brillouin-zone integrations. *J. Mater. Chem. A* **13**, 5188–5192 (1976).

9. Methfessel, M. & Paxton, A. T. High-precision sampling for Brillouin-zone integration in metals. *Phys. Rev. B* **40**, 3616–3621 (1989).

10. Chiarello, G. L., Nachtegaal, M., Marchionni, V., Quaroni, L. & Ferri, D. Adding diffuse reflectance infrared Fourier transform spectroscopy capability to extended x-ray-absorption fine structure in a new cell to study solid catalysts in combination with a modulation approach. *Rev. Sci. Instrum.* **85**, 074102 (2014).

11. Hermans, I. Applications of Modulation Excitation Spectroscopy in Heterogeneous Catalysis. *Ind. Eng. Chem. Res.* **56**, 1123–1136 (2017).

12. Rehr, J. J., Kas, J. J., Vila, F. D., Prange, M. P. & Jorissen, K. Parameter-free calculations of X-ray spectra with FEFF9. *Phys. Chem. Chem. Phys.* **12**, 5503–5513 (2010).

13. Zhang, M., Wang, M., Xu, B. & Ma, D. How to Measure the Reaction Performance of Heterogeneous Catalytic Reactions Reliably. *Joule* **3**, 2876–2883 (2019).

14. White, W. B., Johnson, S. M. & Dantzig, G. B. Chemical equilibrium in complex mixtures. *J. Chem. Phys.* **28**, 751–755 (1958).

15. Tang, C. W., Wang, C. Bin & Chien, S. H. Characterization of cobalt oxides studied by FT-IR, Raman, TPR and TG-MS. *Thermochim. Acta* **473**, 68–73 (2008).

16. Xu, Y. *et al.* Resolving a Decade-Long Question of Oxygen Defects in Raman Spectra of Ceria-Based Catalysts at Atomic Level. *J. Phys. Chem. C* **123**, 18889–18894 (2019).

17. Subramanian, M., Vijayalakshmi, S., Venkataraj, S. & Jayavel, R. Effect of cobalt doping on the structural and optical properties of TiO_2 films prepared by sol-gel process. *Thin Solid Films* **516**, 3776–3782 (2008).

18. Sadezky, A., Muckenhuber, H., Grothe, H., Niessner, R. & Pöschl, U. Raman microspectroscopy of soot and related carbonaceous materials: Spectral analysis and structural information. *Carbon N. Y.* **43**, 1731–1742 (2005).

19. Tsakoumis, N. E., Rønning, M., Borg, Ø., Rytter, E. & Holmen, A. Deactivation of cobalt based Fischer-Tropsch catalysts: A review. *Catal. Today* **154**, 162–182 (2010).

20. Moodley, D. J. *et al.* Carbon deposition as a deactivation mechanism of cobalt-based Fischer-Tropsch synthesis catalysts under realistic conditions. *Appl. Catal. A Gen.* **354**, 102–110 (2009).

21. ten Have, I. C. *et al.* Development of Molybdenum Phosphide Catalysts for Higher Alcohol Synthesis from Syngas by Exploiting Support and Promoter Effects. *Energy Technol.* **7**, 1801102 (2019).

22. Khodakov, A. Y., Chu, W. & Fongarland, P. Advances in the development of novel cobalt Fischer-Tropsch catalysts for synthesis of long-chain hydrocarbons and clean fuels. *Chem. Rev.* **107**, 1692–1744 (2007).

23. Ernst, B., Libs, S., Chaumette, P. & Kiennemann, A. Preparation and characterization of Fischer – Tropsch active Co/SiO_2 catalysts. *Appl. Catal. A Gen.* **186**, 145–168 (1999).

24. Quindimil, A. *et al.* Effect of metal loading on the CO_2 methanation: A comparison between alumina supported Ni and Ru catalysts. *Catal. Today* **356**, 419–432 (2020).

25. Koo, K. Y., Lee, S. H., Jung, U. H., Roh, H. S. & Yoon, W. L. Syngas production via combined steam and carbon dioxide reforming of methane over Ni-Ce/$MgAl_2O_4$ catalysts with enhanced coke resistance. *Fuel Process. Technol.* **119**, 151–157 (2014).

26. Kriesel, J. W. & Tilley, T. D. A new molecular precursor for magnesia-silica materials. *J. Mater. Chem.* **11**, 1081–1085 (2001).

27. Prymak, I., Kalevaru, V. N., Wohlrab, S. & Martin, A. Continuous synthesis of diethyl carbonate from ethanol and CO_2 over Ce-Zr-O catalysts. *Catal. Sci. Technol.* **5**, 2322–2331 (2015).

28. Martin, D. & Duprez, D. Mobility of surface species on oxides. 1. isotopic exchange of $^{18}O_2$ with ^{16}O of SiO_2, Al_2O_3, ZrO_2, MgO, CeO_2, and CeO_2-Al_2O_3. activati. *J. Phys. Chem.* **100**, 9429–9438 (1996).

29. Kydd, R., Teoh, W. Y., Scott, J., Ferri, D. & Amal, R. Probing surface properties and reaction intermediates during heterogeneous catalytic oxidation of acetaldehyde. *ChemCatChem* **1**, 286–294 (2009).

30. Lavalley, J. C. Infrared spectrometric studies of the surface basicity of metal oxides and zeolites using adsorbed probe molecules. *Catal. Today* **27**, 377–401 (1996).

31. Tomishige, K. *et al.* Catalytic performance and properties of ceria based catalysts for cyclic carbonate synthesis from glycol and carbon dioxide. *Green Chem.* **6**, 206–214 (2004).

32. de la Peña O'Shea, V. A., González, S., Illas, F. & Fierro, J. L. G. Evidence for spontaneous CO_2 activation on cobalt surfaces. *Chem. Phys. Lett.* **454**, 262–268 (2008).

33. Pang, X. Y., Liu, C., Li, D. C., Lv, C. Q. & Wang, G. C. Structure sensitivity of CO oxidation on Co_3O_4: A DFT study. *ChemPhysChem* **14**, 204–212 (2013).

34. Nie, X. *et al.* DFT insight into the support effect on the adsorption and activation of key species over Co catalysts for CO_2 methanation. *J. CO2 Util.* **24**, 99–111 (2018).

35. Österlund, L. Structure-Reactivity Relationships of Anatase and Rutile TiO_2 Nanocrystals Measured by In Situ Vibrational Spectroscopy. *Solid State Phenom.* **162**, 203–219 (2010).

36. Chen, W., Zijlstra, B., Filot, I. A. W., Pestman, R. & Hensen, E. J. M. Mechanism of Carbon Monoxide Dissociation on a Cobalt Fischer–Tropsch Catalyst. *ChemCatChem* **10**, 136–140 (2018).

37. Vrijburg, W. L. *et al.* Ni-Mn catalysts on silica-modified alumina for CO_2 methanation. *J. Catal.* **382**, 358–371 (2020).

38. Aguirre, A. & Collins, S. E. Selective detection of reaction intermediates using concentration-modulation excitation DRIFT spectroscopy. *Catal. Today* **205**, 34–40 (2013).

39. Paredes-nunez, A. *et al.* CO Hydrogenation on Cobalt-Based Catalysts : Tin Poisoning Unravels CO in Hollow Sites as a Main Surface Intermediate. *Angew. Chem. Int. Ed.* **130**, 556–559 (2018).

40. Vrijburg, W. L. *et al.* Efficient Base-Metal NiMn/TiO_2 Catalyst for CO_2 Methanation. *ACS Catal.* **9**, 7823–7839 (2019).

41. Hartman, T., Geitenbeek, R. G., Whiting, G. T. & Weckhuysen, B. M. Operando monitoring of temperature and active species at the single catalyst particle level. *Nat. Catal.* **2**, 986–996 (2019).

42. Li, X. *et al.* Controlling CO_2 Hydrogenation Selectivity by Metal-Supported Electron Transfer. *Angew.Chem. Int. Ed.* **59**, 19983–19989 (2020).

43. Smith, E. L. & Porter, M. D. Structure of monolayers of short chain n-alkanoic acids ($CH_3(CH_2)nCOOH$, n = 0-9) spontaneously adsorbed from the gas phase at silver as probed by infrared reflection spectroscopy. *J. Phys. Chem.* **97**, 8032–8038 (1993).

44. Coates, J. Interpretation of Infrared Spectra, A Practical Approach. in *Encyclopedia of analytical chemistry: applications, theory and instrumentation* (ed. Meyers, R. A.) 10815–10837 (John Wiley & Sons, Ltd, 2006).

45. He, Z. *et al.* Synthesis of liquid fuel via direct hydrogenation of CO_2. *Proc. Natl. Acad. Sci. U. S. A.* **116**, 12654–12659 (2019).

46. Lin, L. *et al.* In Situ Characterization of Cu/CeO_2 Nanocatalysts for CO_2 Hydrogenation: Morphological Effects of Nanostructured Ceria on the Catalytic Activity. *J. Phys. Chem. C* **122**, 12934–12943 (2018).

47. Guo, Y. *et al.* Low-Temperature CO_2 Methanation over CeO_2-Supported Ru Single Atoms, Nanoclusters, and Nanoparticles Competitively Tuned by Strong Metal-Support Interactions and H-Spillover Effect. *ACS Catal.* **8**, 6203–6215 (2018).

48. Zhang, Z. *et al.* Support-dependent rate-determining step of CO_2 hydrogenation to formic acid on metal oxide supported Pd catalysts. *J. Catal.* **376**, 57–67 (2019).

49. Mutschler, R., Moioli, E. & Züttel, A. Modelling the CO_2 hydrogenation reaction over Co, Ni and Ru/Al_2O_3. *J. Catal.* **375**, 193–201 (2019).
